# New tribal placement and review of *Parapucaya* Prell and *Pucaya* Ohaus (Coleoptera, Scarabaeidae, Dynastinae)

**DOI:** 10.3897/zookeys.805.28524

**Published:** 2018-12-11

**Authors:** Aura Paucar-Cabrera, Matthew Robert Moore

**Affiliations:** 1 Research Associate, University of Nebraska State Museum, W436 Nebraska Hall, Lincoln, NE 68588-0514, USA University of Nebraska State Museum Lincoln United States of America; 2 Research Associate, Museo QCAZ-Invertebrados, Escuela de Ciencias Biológicas, Pontificia Universidad Católica del Ecuador, Av. 12 de octubre 1076 y Roca, Aptdo. 17-01-2184, Quito, Ecuador Pontificia Universidad Católica del Ecuador Quito Ecuador; 3 Department of Entomology and Nematology, University of Florida, 1881 Natural Drive Area, Steinmetz Hall, Gainesville, FL 32611-0620, USA University of Florida Gainesville United States of America; 4 Florida Department of Agriculture and Consumer Services, Division of Plant Industry, 1911 SW 34th Street, Gainesville, FL 32608, USA Florida Department of Agriculture and Consumer Services Gainesville United States of America

**Keywords:** Cyclocephalini, molecular analysis, morphology, Neotropical scarabs, Pentodontini, taxonomy

## Abstract

The dynastine scarab genera *Parapucaya* Prell and *Pucaya* Ohaus have been historically classified in Pentodontini; however, that tribal classification is not supported under the current tribal circumscriptions. A discussion justifying the transfer of the genera *Parapucaya* and *Pucaya* from Pentodontini into Cyclocephalini is presented. This research is based on morphological observations (mandible shape and wing characters among others) and molecular data (genes 28S, COI, and 16S/ND1). A review of both genera is included, providing descriptions, diagnoses, distribution data, illustrations, and keys to species. A revised key to the world genera of Cyclocephalini is also included.

## Abstract (es)

Los escarabajos de los géneros *Parapucaya* Prell y *Pucaya* Ohaus, de la subfamilia Dynastinae, han sido históricamente clasificados en la tribu Pentodontini; sin embargo, esa clasificación no se ajusta a la circunscripción actual de la tribu. Se presenta una discusión justificando la transferencia de los géneros *Parapucaya* y *Pucaya* de Pentodontini a Cyclocephalini. Esta investigación se basa en observaciones morfológicas (forma de la mandíbula y caracteres del ala membranosa entre otras) y en datos moleculares (genes 28S, COI y 16S/ND1). Se incluye una revisión general de ambos géneros, con descripciones, diagnosis, datos de distribución, ilustraciones, y claves para especies. También se incluye una clave revisada para todos los géneros de Cyclocephalini.

## Introduction

Dynastinae is classified in the scarab beetle family Scarabaeidae, a large coleopteran family that comprises about 30,000 species (Ratcliffe and Cave 2015). Though Scarabaeidae is well-studied, almost 200 new species are described each year (Ratcliffe and Cave 2015). Some adults of Scarabaeidae stand out due to their relatively large size, bright colors, elaborate ornamentation, unique life histories, and many interesting adaptations (Jameson 1998). These exaggerated features are common in the subfamily Dynastinae, which includes about 1,500 species distributed worldwide (Ratcliffe and Cave 2017). More dynastine species are found in the Neotropics than in any other biogeographic realm (Ratcliffe and Cave 2015). In the Neotropics, six of the eight recognized dynastine tribes are represented: Cyclocephalini, Pentodontini, Oryctini, Phileurini, Agaocephalini, and Dynastini. The Neotropical genera *Parapucaya* and *Pucaya* have long been classified in the tribe Pentodontini based on morphological characters, but some recent authors have questioned their tribal placement (Clark 2011, López-García et al. 2015). In this study, we address the classification of *Parapucaya* and *Pucaya* within Pentodontini and redefine the tribe Cyclocephalini.

### Cyclocephalini

**Figures 1–2.**
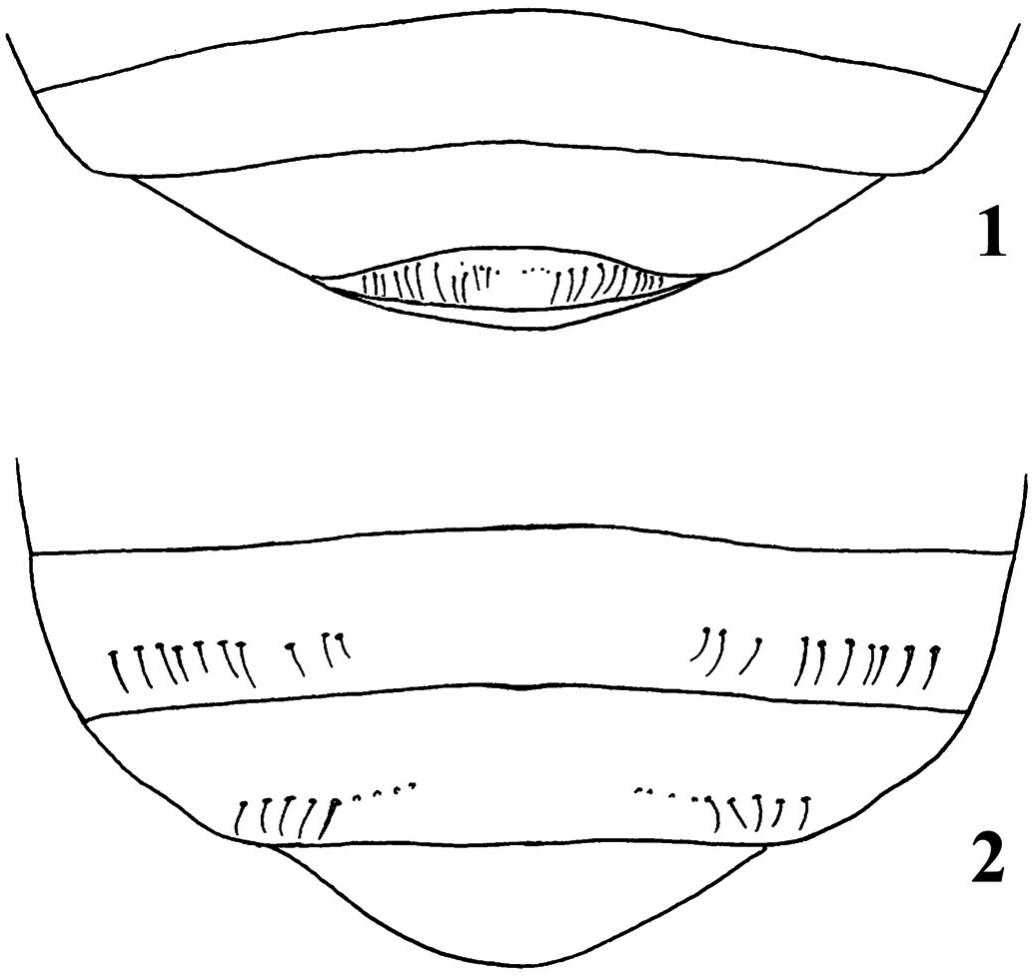
Last sternite in Dynastinae. **1** male with apex emarginate **2** female with apex entire.

Cyclocephalini is the second most species-rich tribe of Dynastinae after Pentodontini, and it contains 14 genera and over 500 species and subspecies (Smith 2006, Moore et al. 2015, 2018b, Ratcliffe and Cave 2017). Historically, the tribe Cyclocephalini was characterized by the absence of characters found in other dynastines. These characters included: 1) lack of horns, tubercles, carinae, or foveae on the head and prothorax; 2) absence of a stridulatory area (*pars stridens*) on the propygidium; 3) simple mandibles that lack dentition distal to the molar region; 4) metatibial apex truncate and without produced teeth or a crenulated margin; and 5) metatarsus with basal joint simple and not triangular (Ratcliffe and Cave 2017). The sexual dimorphism found in cyclocephalines is not as pronounced as it is in the horned dynastines. However, most cyclocephaline species display sexual dimorphism of the protarsus (enlarged in males; simple in females) and elytral epipleural margin (simple in males; expanded and modified in females of some species). Moore (2012) hypothesized that during mating, there was an interaction between the enlarged male protarsal claw and the female epipleural expansion, making it easier for the male to clasp the female during copulation and for mate guarding. Moreover, as in all dynastines, the apex of the last abdominal sternite is emarginate in males and entire or rounded in females (Figs 1, 2).

Cyclocephalini, while relatively morphologically uniform, is not well defined, and monophyly of the tribe still needs to be evaluated (Ratcliffe and Cave 2017). In that endeavor, some generic-level taxa have been removed from the tribe while others have been transferred into Cyclocephalini. *Coscinocephalus* Prell was transferred from Cyclocephalini to Pentodontini and is considered to be most similar to *Orizabus* Fairmaire (Morón and Ratcliffe 1996). The bizarre, monotypic genus *Acrobolbia* Ohaus was transferred from Rutelinae to Cyclocephalini by Jameson et al. (2002), and those authors compared the genus to *Ancognatha* Erichson. *Peltonotus* Burmeister, with 25 species, was transferred from Rutelinae to Dynastinae (Jameson 1998) and specifically to Cyclocephalini (Jameson and Jakl 2010). Additionally, the monophyly of several cyclocephaline genera is in doubt. Ratcliffe (2003) stressed that further research is needed on the genera *Cyclocephala* Dejean, *Mimeoma* Casey, *Aspidolea* Bates, and *Ancognatha* to ascertain if they should be maintained as valid genera or some should be folded into others. Moore et al. (2015) evaluated the monophyly of *Mimeoma* and its relationship with *Cyclocephala* by using a combined molecular and morphological analysis. These data showed that the five species of *Mimeoma* clustered within an apical clade of other *Cyclocephala* species, rendering *Cyclocephala* paraphyletic. As a result, *Mimeoma* was synonymized with *Cyclocephala*.

### Pentodontini

Pentodontini is the largest tribe of dynastines, comprising about 100 genera and over 550 species distributed worldwide (Ratcliffe and Morón 1997, Ratcliffe and Cave 2017). Adult pentodontines are distinguished by: 1) the presence of tubercles, a carina, or a fovea on the head and pronotum; 2) broad mandibles with or without teeth on the scissorial region; 3) propygidium with or without a *pars stridens*; 4) lateral margin of the protibia usually tridentate; 5) apex of the metatibia usually truncate and margined with short, spine-like setae; 6) protarsus occasionally enlarged in males (Ratcliffe and Morón 1997, Ratcliffe and Cave 2017).

Dimorphism between males and females is slight in most species (Ratcliffe and Morón 1997), although males sometimes have larger protarsi and tubercles on the head and pronotum (López-García et al. 2015), and the pronotal fovea is more pronounced. Pentodontines, along with all dynastines, display sexual dimorphism of the last abdominal sternite, which is emarginate in males and entire or rounded in females (Figs 1, 2).

López-García et al. (2015) reported that for over 100 years, there was no consensus whether Pentodontini should be treated as a family, subfamily, or tribe. Historical workers prioritized different criteria: Mulsant (1842) considered the categories Pentodonaires and Oryctésaires as separate groups; followed by Bates (1888) who designated Pentodontinae as a subfamily (Ratcliffe and Morón 1997, López-García et al. 2015); Casey (1915) established Pentodontini as a tribe and later Leng (1920), Arrow (1937), Blackwelder (1944), and Arnett (1973) did not recognize any of these former designations and included pentodontine genera within Oryctini (Ratcliffe and Morón 1997). Endrődi (1969) re-established the tribes Pentodontini and Oryctini as they are currently used, but he considered that transitional characters blurred the distinction between the tribes. Consequently, the monophyly of Pentodontini is in doubt (Ratcliffe 2003, Gasca-Álvarez et al. 2008, López-García et al. 2015, Sanabria-García et al. 2012).

## Materials and methods

### Morphological methods

Morphological descriptions and temporal and distributional data were based on the study of 425 specimens from three sources: (1) field collecting expeditions by the authors and colleagues; (2) data recorded from the literature; and (3) specimens from the following museums and private collections: Canadian Museum of Nature (Ottawa, Canada), Canadian National Collection of Insects (Ottawa, Canada), Florida State Collection of Arthropods (Gainesville, Florida, United States), Museo Ecuatoriano de Ciencias Naturales (Quito, Ecuador), Museo de la Escuela Politécnica Nacional (Quito, Ecuador), National Museum of Natural History (Prague, Czech Republic), Museo QCAZ-Invertebrados de la Pontificia Universidad Católica del Ecuador (Quito, Ecuador), Stephane Le Tirant Collection (Terrabonne, Québec, Canada), University of Nebraska State Museum (Lincoln, Nebraska, United States), and United States National Museum (Washington, DC, currently on long-term loan to University of Nebraska State Museum, Lincoln, Nebraska, United States).

This study was developed as part of the broader project “The Dynastine Scarab Beetles of Ecuador”. For this reason, we provide only generalized, province-level distribution data for *Pucaya* and *Parapucaya* species in Ecuador. More detailed distribution data for these genera will be released as part of that forthcoming monograph. Collecting methods utilized were: 1) light traps using mercury vapor and ultraviolet bulbs; 2) foliage gleaning; 3) excavating rotting logs and stumps; and 4) manual collecting around public lights. Ecuadorian collecting, mobilization, and export permits were obtained with the support of QCAZ in Quito, Ecuador.

The species descriptions encompass the range of variation observed in the specimens at hand. They were based on the following characteristics (from Ratcliffe and Cave 2017): 1) length from apex of the clypeus to the apex of the elytra; 2) width across elytral humeri; 3) coloration and markings; 4) interocular width (number of transverse eye diameters across the frons between the eyes); 5) form and sculpturing of the head, pronotum, elytra, and pygidium; 6) form of the prosternal process; and 7) form of the parameres. Punctures were considered simple unless otherwise noted. Minute punctures were generally not visible with 12.5× magnification but were easily seen with 50× magnification. Small punctures were clearly visible with 12.5× magnification and can be seen with the naked eye. Large punctures are easily seen without magnification. Sparse punctures were characterized by greater than 5 puncture diameters between them. Punctures moderate in density had 3–5 puncture diameters between them. Dense punctures had only 2 or fewer puncture diameters between them.

### DNA extraction, PCR, and data-mining

Previous studies by Gunter et al. (2016) and Ahrens et al. (2011, 2014) generated DNA sequence data that served as a phylogenetic scaffold for testing the classification of *Pucaya* and *Parapucaya* within Pentodontini (Tab. 1). GenBank was datamined for 28S, CO1, and 16S/ND1 sequences from diverse tribal-level exemplars for higher Scarabaeidae (Tab. 1). Among Dynastinae, there were tribal-level exemplars with at least partial data for all three gene regions from six of the eight commonly recognized tribes (minus Hexodontini and Agaocephalini). 16S and 28S data were generated from exemplar specimens of *Pucayapulchra* Arrow and *Parapucayaamazonica* Prell to incorporate into this phylogenetic framework. Based on shared morphological characters with *Pucaya* species, *Cyclocephalafreyi* Endrődi exemplars were also targeted for DNA extraction and PCR.

DNA extractions of metafemoral tissue from specimens of *C.freyi*, *P.amazonica*, and *P.pulchra* were performed using guanidinium thiocyanate following the QCAZ Molecular Biology Laboratory protocol (unpublished). 28S sequence data was gathered using the primers Bulbasaur/28SR (Moore et al. 2015, Whiting et al. 1997, Whiting 2001) and thermocycles from Moore et al. (2015). 16S sequence data was generated using the universal primer LRJ-12864 and 16Sar-L (Palumbi et al. 1991) with the following thermocycle: 1) 94 °C for 2 minutes; 2) 94 °C for 40 seconds; 3) 54 °C for 40 seconds; 4) 68 °C for 1 minute and 30 seconds (34 cycles of steps 2–4); and a final extension of 68 °C for 1 minute. Forward and reverse sequence traces were trimmed and assembled into contigs in Geneious 5.6.2 (Kearse et al. 2012).

### Alignments and phylogenetic analyses

Based on the results of Gunter et al. (2016), a species of *Isonychus* Mannerheim (Scarabaeidae: Melolonthinae: Macrodactylini) was used as an outgroup for all phylogenetic analyses. *Isonychus* sp. was recovered as the most early-diverging member of a melolonthine clade sister to the clade containing all Cetoniinae + Rutelinae + Dynastinae exemplars (Gunter et al. 2016), making this taxon a suitable outgroup for examining relationships among these subfamilies and placing *Pucaya* and *Parapucaya* at the tribal level. Sequences were aligned using ClustalW (Larkin et al. 2007), with default settings, as implemented in MEGA7 (Kumar et al. 2016). The resulting concatenated sequence alignment contained 3,537 bp positions (1530 bp 16S; 550 bp ND1; 805 bp CO1; 652 bp 28S).

Maximum likelihood analyses of this matrix were conducted in W-IQ-TREE (Trifinopoulos et al. 2016). The matrix was partitioned by gene (16S, 28S, and ND1) and codon position (CO1). The best-fit model of sequence evolution for each partition (GTR+F+I+G4 for 16S; TPM3u+F+G4 for ND1 and CO1 third position; TIM2e+I+G4 for 28S; HKY+F+I+G4 for CO1 first position; SYM+I+G4 for CO1 second position) was selected by ModelFinder (Kalyaanamoorthy et al. 2017), as implemented in W-IQ-TREE, using the Bayesian information criterion. Bootstrap support values for the most likely tree were calculated using 10,000 ultrafast bootstrap replicates (Hoang et al. 2017). Bayesian phylogenetic analyses were run in MrBayes 3.2.2 (Ronquist and Huelsenbeck 2003). Models of sequence evolution for the MrBayes analysis were determined with PartitionFinder 2.1.1 (GTR+I+G for 16S, ND1, 28S, CO1 second position, and CO1 third position; HKY+I+G for CO1 first position) (Lanfear et al. 2016).

Bayesian analyses comprised four independent runs, each with four chains (one cold and three heated). Partitions had their parameters unlinked and allowed to vary independently. Flat priors were used. Chains were run for 1 million generations, with trees sampled every 1,000 generations. Convergence was evaluated by examining the standard deviation of split frequencies among runs and by plotting the log-likelihood values from each run using Tracer 1.6 (Rambaut and Drummond 2013). Tracer diagnostics indicated that runs converged within 10,000 generations, and trees sampled during this period were discarded as burn-in before obtaining clade posterior probabilities. Parsimony tree searches were performed in MPBoot (Hoang et al. 2018). Heuristic searches were conducted using default parsimony ratchet search options in MPBoot. Bootstrap analyses were performed using the same ratchet search options and included 10,000 bootstrap replicates.

**Table 1.** GenBank accession numbers of the taxa analyzed in this study. Molecular sequences of *Cyclocephalafreyi* Endrődi, *Parapucayaamazonica* Prell, and *Pucayapulchra* Arrow included in this study were obtained from the Museo de Zoología QCAZ, at the Pontificia Universidad Católica del Ecuador in Quito.

Taxa	28S accessions	CO1 accessions	16S/ND1 accessions
**Melolonthinae: Macrodactylini**			
*Isonychus* sp.	HQ599181	HQ599132	HQ711606
**Cetoniinae: Cetoniini**			
*Chilolobaacuta* (Wiedemann)	DQ524778	DQ524540	DQ680981
*Glycyphana* sp.	KF802022	KF801859	KF801691
*Heterocnemisgraeca* Brulle	EU084147	EU084042	EF487942
*Protaetia* sp.	KF802102	KF801937	KF801775
**Cetoniinae: Goliathini**			
*Heterorrhinamicans* (Guérin Méneville)	DQ524738	DQ524507	DQ681041
**Cetoniinae: Schizorhinini**			
*Bisallardiana* sp.	KF802033	KF801870	KF801702
*Chlorobapta* sp.	KF802101	KF801935	KF801773
*Chondropyga* sp.	KF802038	KF801875	KF801707
*Dilochrosis* sp.	KF802056	KF801891	KF801727
*Eupoecila* sp.	KF802032	KF801869	KF801701
*Hemipharis* sp.	KF802088	KF801924	KF801760
*Lomaptera* sp.	KF802099	KF801933	KF801771
*Lyraphora* sp.	KF802058	KF801893	KF801729
*Mycterophallus* sp.	KF802134	KF801970	KF801806
*Pseudoclithria* sp.	KF802100	KF801934	KF801772
*Trichaulax* sp.	KF802053	KF801888	KF801724
**Dynastinae: Cyclocephalini**			
*Cyclocephalafreyi* Endrődi	MH938363	–	MH938360
*Cyclocephala* sp.	JN969246	JN969202	EF487979
*Cyclocephala* sp.	HQ599137	HQ599096	HQ711605
*Cyclocephala* sp.	HQ599138	HQ599097	HQ711596
**Dynastinae: Dynastini**			
*Xylotrupes* sp.	KF802040	KF801877	KF801709
**Dynastinae: Oryctini**			
*Oryctesnasicornis* (Linnaeus)	JN969247	EF487735	EF487922
**Dynastinae: Oryctoderini**			
*Onychionyx* sp.	KF802089	KF801925	KF801761
*Oryctoderus* sp.	KF802090	KF801926	KF801762
**Dynastinae: Pentodontini**			
*Alissonotumbinodulum* Fairmaire	DQ524763	DQ524544	DQ680957
*Alissonotumsimile* Arrow	DQ524584	DQ524481	DQ681016
*Carneodon* sp.	KF802161	KF801998	KF801832
*Cheiroplatys* sp.	KF802054	KF801889	KF801725
*Heteronychuslioderes* Redtenbacher	DQ524753	DQ524542	DQ680955
*Metanastes* sp.	KF802007	KF801841	KF801675
*Neocorynophyllus* sp.	KF802137	KF801973	KF801809
*Novapus* sp.	KF802021	KF801858	KF801690
*Parapucayaamazonica* Prell	MH938364	–	MH938361
*Pentodonidiota* Herbst	EU084151	EU084045	EF487918
*Phyllognathusdionysius* Fabricius	EU084152	EF487737	EF487944
*Pimelopusdubius* Blackburn	JN969249	EF487738	EF487960
*Pucayapulchra* Arrow	MH938365	–	MH938362
*Semanopterus* sp.	KF802008	KF801842	KF801676
*Semanopterus* sp.	KF802075	KF801909	KF801746
*Trissodon* sp.	KF802067	KF801900	KF801738
**Dynastinae: Phileurini**			
*Cryptodus* sp.	KF802020	KF801857	KF801689
*Eophileurus* sp.	KF802057	KF801892	KF801728
**Rutelinae: Adoretini**			
*Adoretuslasiopygus* Burmeister	DQ524794	DQ524555	DQ680980
*Adoretus* sp.	DQ524671	DQ524444	DQ680986
*Adoretus* sp.	DQ524672	DQ524445	DQ680964
*Adoretusversutus* Harold	DQ524766	DQ524450	DQ680948
*Prodoretustruncatus* (Arrow)	EU084292	EU084139	EF487915
*Trigonostomummucoreum* Burmeister	EU084293	EU084140	EF487916
**Rutelinae: Anomalini**			
*Anomalabengalensis* (Blanchard)	DQ524741	DQ524510	DQ680971
*Anomalabiharensis* Arrow	DQ524723	DQ524519	DQ680974
*Anomalabilobata* Arrow	DQ524607	DQ524495	DQ680977
*Anomalapraenitens* Arrow	DQ524792	DQ524553	DQ681042
*Anomalavariegate* Hope	DQ524760	DQ524524	DQ680938
*Blithopertha* sp.	EU084289	EU084137	EF487957
*Isoplialasiosoma* Burmeister	HQ599172	HQ599124	HQ711583
*Mimelasiliguria* (Arrow)	DQ524724	DQ524498	DQ680959
**Rutelinae: Anoplognathini**			
*Anoplognathus* sp.	KF802029	KF801866	KF801698
*Anoplostethus* sp.	KF802160	KF801997	KF801831
*Anoplostethus* sp.	KF802157	KF801994	KF801829
*Calloodes* sp.	KF802091	–	KF801763
*Phalangogoniasperata* Sharp	KJ845157	–	–
*Repsimus* sp.	KF802028	KF801865	KF801697
*Repsimus* sp.	KF802092	KF801927	KF801764
**Rutelinae: Geniatini**			
*Geniates* sp.	HQ599185	–	HQ711603
*Lobogeniates* sp.	HQ599186	–	HQ711604
**Rutelinae: Rutelini**			
*Anticheira* sp.	HQ599184	–	HQ711600
*Parastasia* sp.	KF802096	KF801930	KF801768
*Parastasia* sp.	KF802086	KF801920	KF801757
*Parastasia* sp.	KJ845160	–	–
*Pelidnota* sp.	HQ599187	–	HQ711602

## Results

### Morphology

**Figures 3–4.**
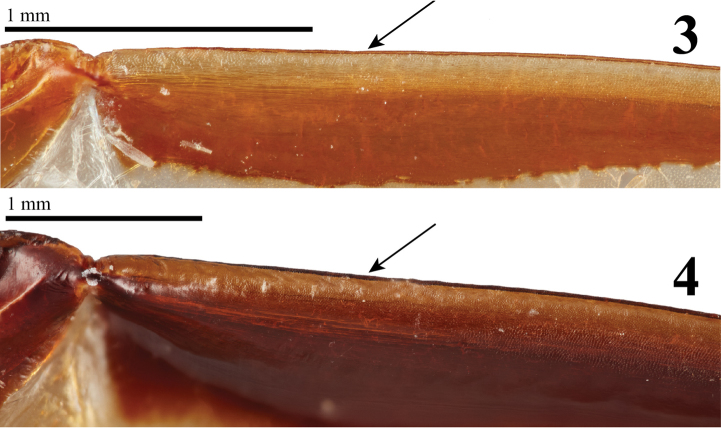
Hindwing vein RA3, distal to apical hinge. **3***Parapucaya* sp. **4***Pucaya* sp. Photo credits to Gavin J. Martin.

Morphological observations show that *Parapucaya* shares characters with genera in Cyclocephalini, most notably with some *Cyclocephala* species. For example, the two *Parapucaya* species share characters with *C.almitana* Dechambre, *C.macrophylla* Erichson, *C.melanocephala* (Fabricius), and *C.pseudomelanocephala* Dupuis. These characters include: 1) frontoclypeal suture complete; 2) clypeus weakly emarginate with lateral and apical margins reflexed; 3) clypeal apex broadly truncate; 4) the generally exposed and slender mandibles that lack lateral teeth; 5) mandibular apex acuminate and curved upward; 6) protibia strongly tridentate with the basal tooth removed from other two teeth; 7) protarsus in males enlarged (the larger claw strongly curved and incised at apex), while females have a simple protarsus; 8) inner portion of the apical margin of the 5^th^ protarsomeres in males eroded, allowing the enlarged protarsal claw to be further articulated; 9) metatarsi reduced, shorter than metatibia, more evident in females (character shared with *C.melanocephala* and *C.almitana*); 10) prosternal process moderately long, columnar, with its apex densely setose, flattened, and with a large, raised, round “button” covering half of the apex; 11) hindwing vein RA, proximal to apical hinge, with 2 rows of pegs extending distally nearly to margin of apical hinge; and 12) anterior edge of hindwing distal to apical hinge lacking setae and with a produced, membranous border (Figs 3, 4).

Like *Parapucaya*, *Pucaya* species share many characters with some *Cyclocephala* species (e.g., *C.freyi*). *Pucaya* also shares the character of a medially incomplete frontoclypeal suture with *Ancognatha* species. In *Pucaya* individuals, the frontoclypeal suture is visible from the lateral margins along the external side of the frontal horn, where it becomes obsolete medially. *Pucaya* and some *Ancognatha* species display weakly developed “armature” of the head and thorax. For example, *Ancognathacastanea* Erichson has tubercle-like swellings on the frontoclypeal region of the head. *Ancognathajamesoni* Murray and *A.horrida* Endrődi show enlargement of the pronotum in males.

**Figures 5–6.**
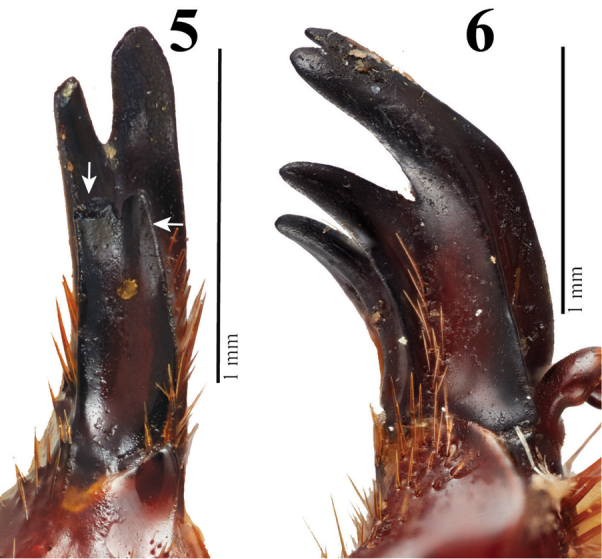
Maxillary galea showing four teeth. **5***Cyclocephalafreyi* Endrődi **6***Pucayapulchra* Arrow. Photograph credits to Gavin J. Martin.

Other shared characters with other cyclocephalines include; 1) clypeus with lateral and apical margins reflexed; 2) clypeal apex broadly truncate, subquadrate; 4) maxillary galea with four teeth on inner margin (shared with *C.freyi* (Figs 5, 60), 5) slender mandibles that lack lateral teeth; 6) protibia strongly tridentate with the basal tooth removed from other two teeth; 7) protarsus in males enlarged (the larger claw strongly curved and incised at apex), while females have a simple protarsus; 8) inner portion of the apical margin of the 5^th^ protarsomeres in males eroded, allowing the enlarged protarsal claw to be further articulated; 9) prosternal process short to moderately long, columnar, with its apex densely setose, flattened, and with a large, raised, round “button” covering half of the apex; 11) hindwing vein RA, proximal to apical hinge, with 2 rows of pegs extending distally nearly to margin of apical hinge; and 12) anterior edge of hindwing distal to apical hinge lacking setae and with a produced, membranous border.

### Molecular phylogenetic analyses

**Figure 7.**
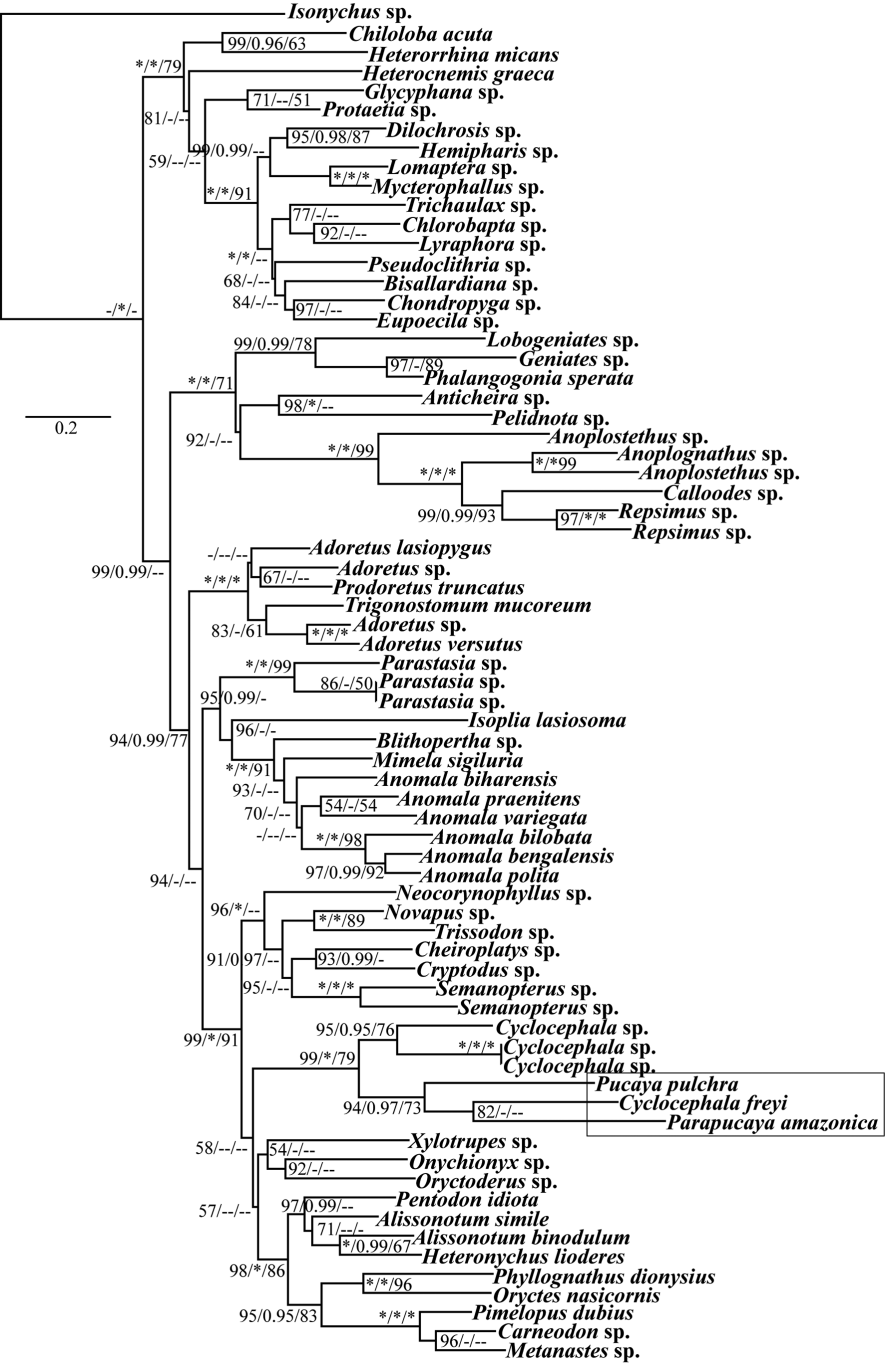
Bootstrap consensus tree from W-IQ-TREE analysis. Node support values from left to right are ML bootstrap, Bayesian posterior probability, and parsimony bootstrap. Support values labeled with a “*” have 100% bootstrap support or 1.0 posterior probability. Support values labeled with a “-” have bootstrap supports lower than 50% or posterior probability lower than 0.95. Nodes labeled “--” indicates that node was not recovered by an analysis.

W-IQ-TREE analyses found the most likely tree with a log likelihood score of -42928.5840. MPBoot heuristic tree searches recovered most parsimonious trees of score 9992 steps. Bayesian posterior probabilities and parsimony bootstrap support values for nodes are reported on the maximum likelihood bootstrap consensus tree topology (Fig. 7). Analyses conducted on the concatenated dataset recovered 27 strongly supported internal nodes (>75 BS and >0.95 PP) from all three tree search strategies. All three analyses strongly supported the monophyly of Cetoniinae and Dynastinae (Fig. 7). Like the analyses of Gunter et al. (2016), the subfamily Rutelinae was recovered as paraphyletic. *Parapucayaamazonica*, *P.pulchra*, and *C.freyi* were recovered together as a clade (94 ML BS, 0.97 PP, 73 Parsimony BS) sister to the other three *Cyclocephala* exemplars. Together, these six exemplars form a strongly supported cyclocephaline clade (99 ML BS, 1.0 PP, 79 Parsimony BS) within the broader Dynastinae clade (99 ML BS, 1.0 PP, 91 Parsimony BS) (Fig. 7). The remaining 14 Pentodontini species included here did not form a monophyletic group. Six pentodontine species fell out in a clade that includes *Cryptodus* sp. (Dynastinae: Phileurini) (96 ML BS, 1.0 PP). Eight pentodontine species were recovered in a clade (98 ML BS, 1.0 PP, 86 Parsimony BS) that also included *Oryctesnasicornis* (Linnaeus) (Dynastinae: Oryctini).

## Discussion

*Parapucaya* and *Pucaya* were placed in Pentodontini by previous authors, and this tribal-level classification has been maintained since Endrődi’s (1985) revision of world Dynastinae. *Parapucaya* and *Pucaya* species were placed in Pentodontini because of their armature, such as the minute tubercles of the pronotum in *Parapucaya* species and the cephalic horns and tubercles of *Pucaya* species. These characters violated the tribal circumscription of Cyclocephalini. However, these two genera also complicate the traditional circumscription of Pentodontini. For example, *Parapucaya* and *Pucaya* have slender mandibles, and males and females can be easily distinguished by external characters.

Based on the morphological observations outlined in the previous section, we think that *Parapucaya* species are most similar to the *C.melanocephala* section of *Cyclocephala*. Additionally, we think that *Pucaya* species are most similar to *C.freyi* based on the shared form of the four-toothed galea present in all these species (Figs 5, 6). The following characters also support the hypothesis that *Pucaya* and *Parapucaya* are cyclocephalines: clypeus with lateral and apical margins reflexed; the clypeal apex broadly truncate shared with several *Cyclocephala* species; mandibles lacking lateral teeth; protibia strongly tridentate with the basal tooth removed from other two apical teeth; protarsus in males enlarged (the larger claw strongly curved and incised at apex), while females have a simple protarsus; and the inner portion of the apical margin of the 5^th^ protarsomeres in males eroded, allowing the enlarged protarsal claw to be further articulated.

Study of the hindwings also showed that *Pucaya* and *Parapucaya* share the same character states: hindwing vein RA, proximal to the apical hinge, with two rows of pegs extending distally nearly to margin of apical hinge and the anterior edge of hindwing distal to apical hinge lacking setae and with a produced, membranous border. This exact combination of hindwing characters is also found in the cyclocephaline genera *Arriguttia* Martínez, *Aspidolea*, *Augoderia* Burmeister, most *Cyclocephala* (except black species formerly placed in *Mononidia* Casey or *Surutoides* Endrődi), and former *Mimeoma* species (Moore et al. 2018a). The genera *Acrobolbia*, *Ancognatha*, and *Ruteloryctes* also share the membranous border on the leading edge of RA3 but lack the double row of pegs on RA (Moore et al. 2018a). No other tribe of Dynastinae shares the character of a membranous border on RA3 (MRM, unpublished data). This hindwing character is a putative synapomorphy uniting these cyclocephaline genera plus *Pucaya* and *Parapucaya*.

Additionally, the molecular phylogenetic analyses presented here also support revised placement of *Pucaya* and *Parapucaya* in Cyclocephalini. Our analyses recovered a monophyletic Dynastinae with strong statistical support (Fig. 7). These analyses also recovered a strongly supported clade that included four *Cyclocephala* exemplars plus *P.castanea* and *P.amazonica* (Fig. 7). We think the weight of evidence supports the hypothesis that *Pucaya* and *Parapucaya* are part of the cyclocephaline lineage of Dynastinae. Based on morphological observations, we also think that *Pucaya* and *Parapucaya* are most likely to be closely related to sections of *Cyclocephala*. Thus, we formally move the genera *Pucaya* and *Parapucaya*, as a revised tribal placement, from Pentodontini into Cyclocephalini.

Historically, Cyclocephalini has been defined by the lack of characters present in other tribes, such as the lack of horns or tubercles, foveae, or carinae. However, this was an inconsistent concept as *Ancognatha* species with weakly developed cephalic and thoracic armature, (e.g., tubercles, enlarged pronotum, and enlarged mandibles) were already classified in Cyclocephalini. This work categorically indicates that Cyclocephalini includes individuals with armature. This is a potentially fascinating re-circumscription of the tribe, as the role of cephalic and thoracic armature is completely unknown for *Pucaya*, *Parapucaya*, and Cyclocephalini more broadly.

## Review of *Parapucaya* Prell and *Pucaya* Ohaus

We present a revised key to the New World Cyclocephalini genera. We include a redescription of the species of *Parapucaya* and *Pucaya*, diagnosis, distribution data, and available natural history information. We include keys to species of both genera.

### Key to the world genera of adult Cyclocephalini

(Modified from Jameson et al. 2002)

*Males*: Apex of last abdominal sternite emarginate (Fig. 1). Protarsomeres 4–5 and/or anterior claw enlarged in all genera except *Stenocrates* and *Erioscelis*.

*Females*: Apex of last abdominal sternite entire, evenly parabolic (Fig. 2). Protarsomeres 4–5 and anterior claw always simple, not enlarged.

**Table:** 

1	Head with small horn or tubercle mesad of each eye (Figs 35, 36). Costa Rica to Ecuador	***Pucaya* Ohaus, 1910**
–	Head without horn or tubercle mesad of each eye (*Ancognathacastanea* Erichson has frons with low, median knob or elevated, transverse tubercle)	**2**
2	Apex of labrum chitinized (thickened). Labrum extends past the apex of the clypeus in dorsal view (Fig. 8). Asia	***Peltonotus* Burmeister, 1847**
–	Apex of labrum not conspicuously thickened. Labrum does not extend past the apex of the clypeus in dorsal view	**3**
3	Mandibles broad, nearly as wide as long (Fig. 9). West Africa	***Ruteloryctes* Arrow, 1908**
–	Mandibles narrow, distinctly longer than wide	**4**
4	Propygidium mostly covered by elytra, with long, dense setae that protrude from beneath elytral apices; propygidium often elongated, so that pygidium appears moderately to extremely shortened. Body noticeably tapered at both ends. Protarsus in males with tarsomeres 4–5 and claw enlarged. South America, West Indies	***Chalepides* Casey, 1915**
–	Propygidium lacking long, dense setae; length of propygidium normal. Body not noticeably tapering at both ends. Protarsus in males with tarsomeres and claw enlarged or not	**5**
5	Body form strongly flattened, relatively large (24–44 mm). Color black. Clypeus with apex narrowly to broadly parabolic (Figs 10, 11)	**6**
–	Body form not flattened, size smaller (6–29 mm, and some larger individuals of *Ancognatha*). Color variable, including patterns. Clypeus with apex variable, parabolic or not	**7**
6	Eyes large, interocular width equals 2.0 or less transverse eye diameters. Males with protibia slender, strongly curved, with distinct tooth on inner margin near base (Fig. 12); anterior trochanter with large, anteriorly projecting tooth. Northern South America	***Harposceles* Burmeister, 1847**
–	Eyes smaller, interocular width usually 3.0 or more transverse eye diameters. Males with protibia “normal”, not curved strongly, lacking tooth on inside near base; anterior trochanter lacking anteriorly projecting tooth. South America	***Surutu* Martínez, 1955**
7	Clypeus with sides slightly wider than base before abruptly narrowing to acuminate apex (Fig. 13). Males with antennal club almost twice as long as antennomeres 1–7 (Fig. 13). Meso- and metatibiae at apex with spinose process on external edge. Northwestern South America	***Acrobolbia* Ohaus, 1912**
–	Clypeus with sides tapering from base to apex (rounded, parabolic, subtriangular, or sharply acuminate), or with sides divergent from base to apex, but with apex never abruptly acuminate (Figs 14–24). Males with antennal club slightly longer than, subequal to, or shorter than antennomeres 2–7. Meso- and metatibiae at apex without spinose process on external edge	**8**
8	Lateral margins of clypeus near base raised into a subacute crest, evident in posterodorsal view (Fig. 25). Clypeus thickened along the frontoclypeal suture. Frontoclypeal disc concave (Fig. 25). Specimens with double tubercles or faint declivity near anterior margin of pronotum. Costa Rica to Peru and Brazil	***Parapucaya* Prell, 1934**
–	Lateral margins of clypeus near base flat or faintly raised into a round crest, evident in posterodorsal view (Fig. 26). Clypeus flat or weakly thickened along the frontoclypeal suture. Frontoclypeal disc convex or concave (Fig. 26). Specimens without double tubercles or faint declivity near anterior margin of pronotum	**9**
9	Clypeus trapezoidal or subtrapezoidal, with marginal or apical bead (Fig. 27–28)	**10**
–	Clypeus with apex rounded, truncate, subquadrate, or emarginate, simple, with or without marginal bead (Figs 14–24)	**11**
10	Frontoclypeal suture distinct, usually broadly depressed just before suture. Males with protarsomeres simple, not enlarged. Pronotum with anterior margin normally arcuate, not produced forward at middle (Fig. 27). Meso- and metafemora and meso- and metatibiae strongly flattened. Central and South America	***Stenocrates* Burmeister, 1847**
–	Frontoclypeal suture a finely impressed line but not with deep and broad impression before it. Males with anterior claw and usually protarsomeres 4–5 enlarged. Pronotum on anterior margin produced anteriorly at middle (Fig. 28). Meso- and metafemora and meso- and metatibiae not strongly flattened. North, Central, and South America, West Indies	***Dyscinetus* Harold, 1869**
11	Body form short, suboval, stout; elytra nearly as wide as long. Clypeus subquadrate, about twice as wide as long, apex broad, subtruncate, broadly reflexed (Fig. 14). Size 14–16 mm. Brazil, French Guiana	***Arriguttia* Martínez, 1960**
–	Body form usually elongate, not short or suboval or stout; if so, then length less than 14 mm (usually 9–12 mm). Clypeus with apex rounded, broadly parabolic, subquadrate, or emarginate (Figs 15–24)	**12**
12	Clypeus with sides usually divergent (sometimes only slightly) from base to apex, apex broadly rounded (Fig. 16). Maxilla lacking well-developed teeth (when teeth present they are minute and obscured by setae), apex penicillate (setae usually long and dense). Mexico to Argentina	***Aspidolea* Bates, 1888**
–	Clypeus with sides parallel or convergent from base to apex (never divergent), apex rounded, subtruncate, or emarginate. Maxilla armed with distinct teeth, apex rarely penicillate (a few species of *Cyclocephala*)	**13**
13	Elytra distinctly, irregularly punctate, punctures not in regular rows; surface with or without weak metallic sheen. Clypeus with apex nearly semicircular, margin beneath apex distinctly thickened (Fig. 15). Venezuela, Peru, Bolivia, Brazil	***Augoderia* Burmeister, 1847**
–	Elytra smooth or distinctly punctate, some punctures in regular rows; surface never with metallic sheen. Clypeus with apex variable, semicircular or not, margin beneath apex not distinctly thickened	**14**
14	Clypeus subquadrate, sides weakly converging to broad apex, apex truncate or emarginate (Figs 17, 18). Interocular width 6.0 or more transverse eye diameters (Figs 17, 18). Males protarsus and claw simple, not enlarged. Central and South America	***Erioscelis* Burmeister, 1847**
–	Clypeus not subquadrate, instead with sides converging from base to rounded, parabolic, subtriangular, or emarginate apex (Figs 19–24). Interocular width 5.0 or less transverse eye diameters (Figs 19–24). Males protarsus enlarged, with bifurcate median claw	**15**
15	Mentum with apex distinctly (often deeply) emarginate, surface at center furrowed in apical third (Fig. 29). Mandible elongated, apex extended to or beyond clypeal apex. Frontoclypeal suture obsolete medially. Length usually more than 18 mm, rarely as small as 15 mm. North, Central, and South America	***Ancognatha* Erichson, 1847**
–	Mentum with apex truncate or weakly emarginate (Fig. 30). Mandibles not elongated. Frontoclypeal suture more or less complete. Length variable, 6–35 mm. Canada to Argentina, West Indies (one species introduced to Australia, one species introduced to Hawaii)	***Cyclocephala* Latreille, 1829**

**Figures 8–15.**
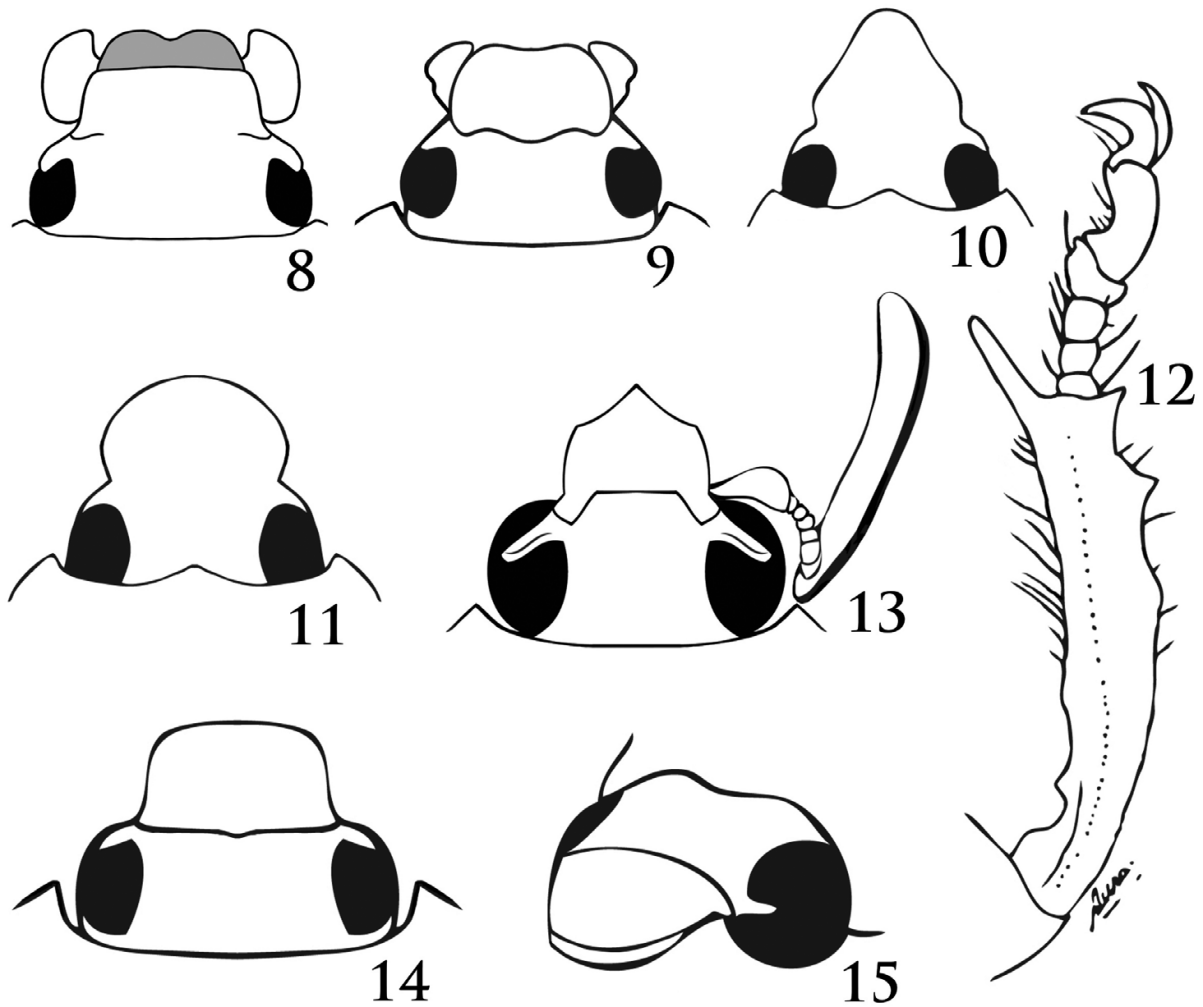
Form of clypeal apex. **8***Peltonotussilvanus* Jameson and Wada (subquadrate, note chitinized labrum) **9***Ruteloryctesmorio* (Fabricius) (emarginate, note round mandibles) **10***Surutuseabrai* d’Andretta and Martínez (narrowly parabolic) **11***S.hesperius* Ratcliffe (broadly parabolic) **12** Protibia of *Harposceles* sp. **13***Acrobolbia* sp. (pentagonal) **14***Arriguttia* sp. **15***Augoderia* sp. (thickened apex) (Figures 9–15 modified from Jameson et al. 2002, by permission).

**Figures 16–24.**
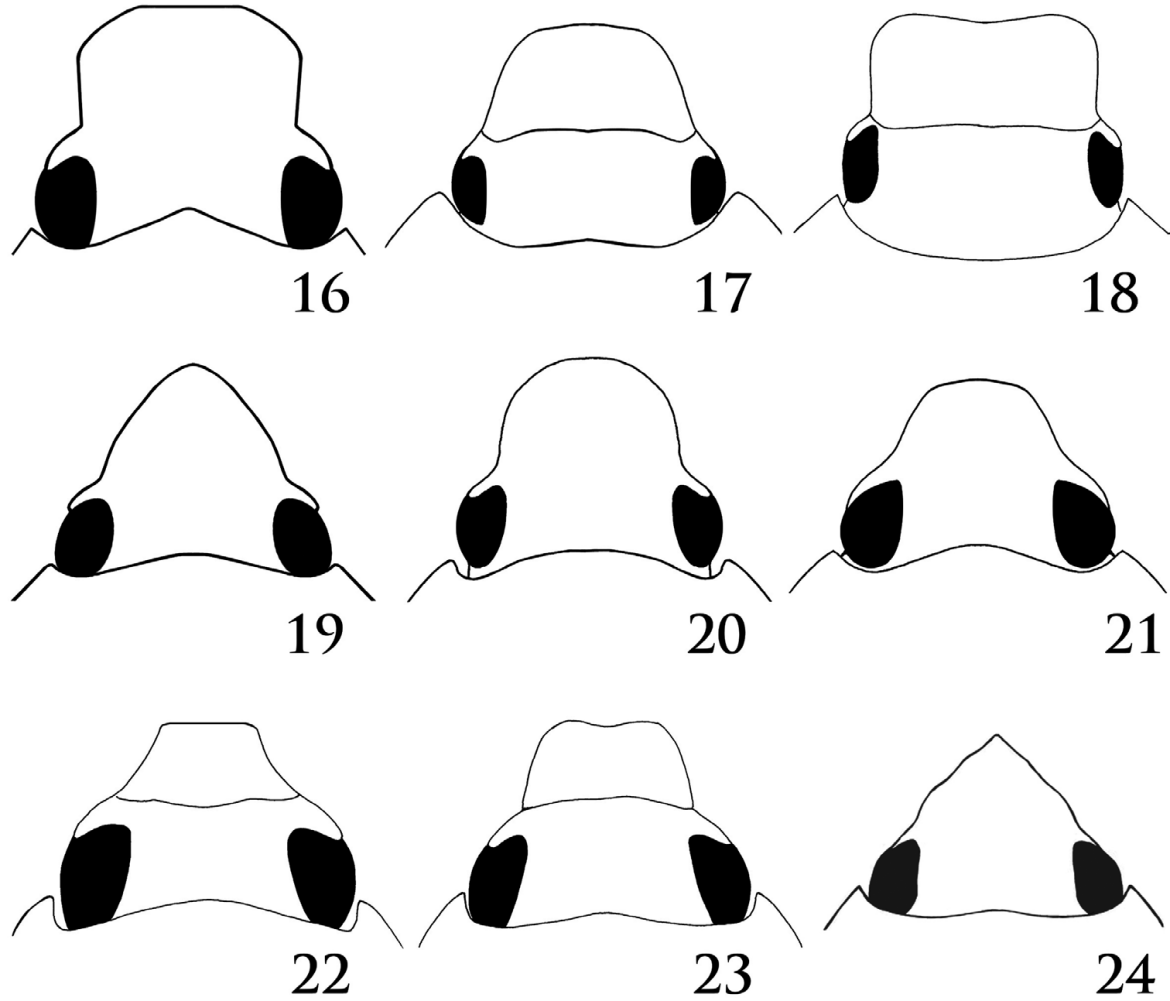
Form of clypeal apex. **16***Aspidolea* sp. (subquadrate, dirvergent from base to apex) **17***Erioscelis* sp. (subquadrate truncate) **18***Erioscelis* sp. (subquadrate emarginate) **19***Ancognatha* sp. (narrowly parabolic) **20***Ancognatha* sp. (parabolic) **21***Cyclocephala* sp. (rounded) **22***Cyclocephala* sp. (truncate) **23***Cyclocephala* sp. (emarginate) **24***Cyclocephala* sp. (sharply acuminate) (Figures modified from Jameson et al. 2002 and Ratcliffe 2003, by permission).

**Figures 25–30.**
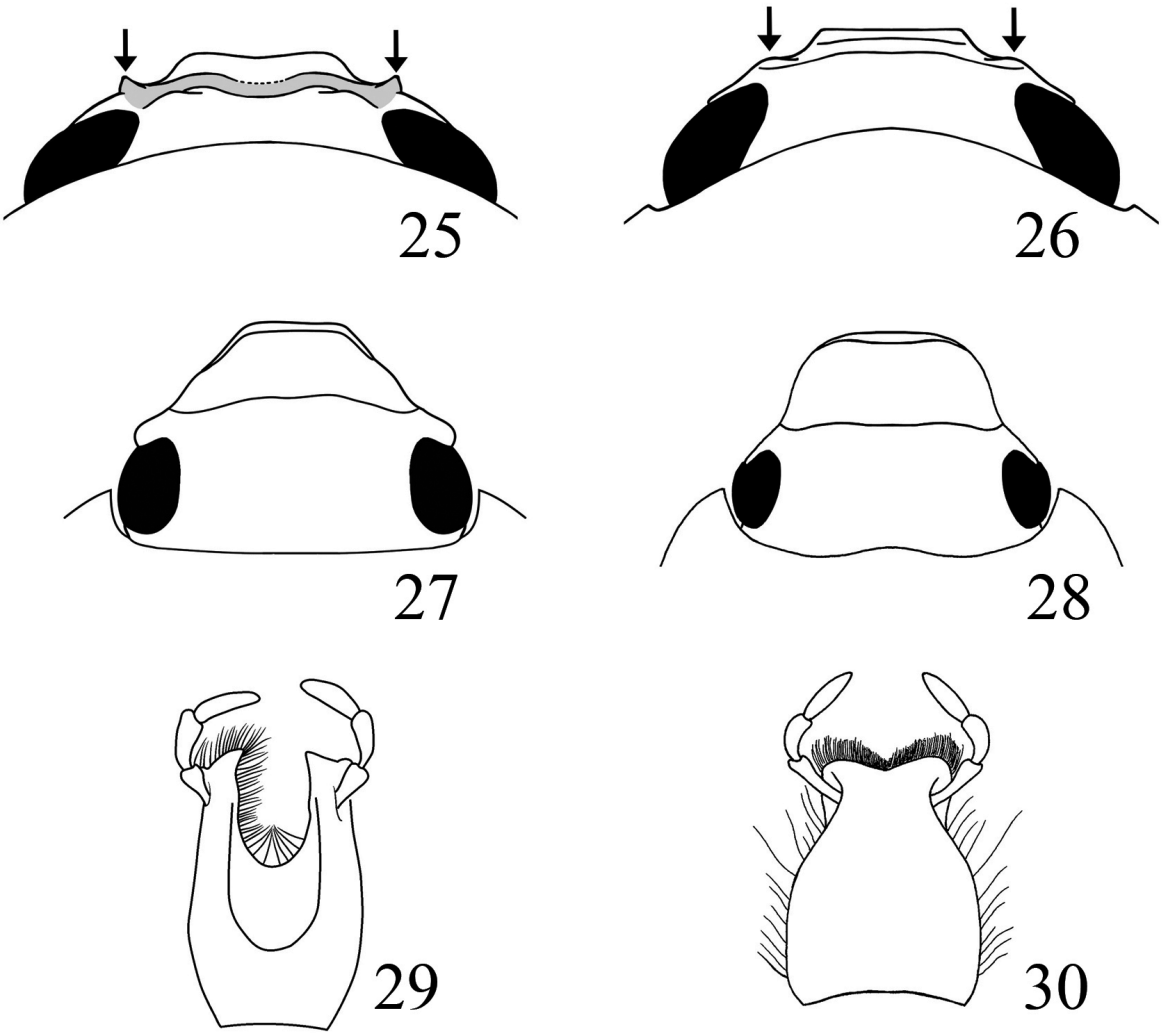
Form of clypeus and pronotum. **25***Parapucaya* sp. (clypeus thickened with sharp crest on margins) **26***Cyclocephalamelanocephala* (Fabricius) (clypeus not thickened, margins weakly raised, rounded). **27***Stenocrates* sp. (clypeus trapezoidal, pronotum not produced anteriorly at middle) **28***Dyscinetus* sp. (clypeus subtrapezoidal, pronotum with anterior margin produced at middle). Form of clypeus in posterodorsal view Mentum **29***Ancognatha* sp. (with furrow) **30***Cyclocephala* sp. (without furrow, weakly emarginate). (figures 28–30 from Ratcliffe 2003, by permission).

**Figure 31.**
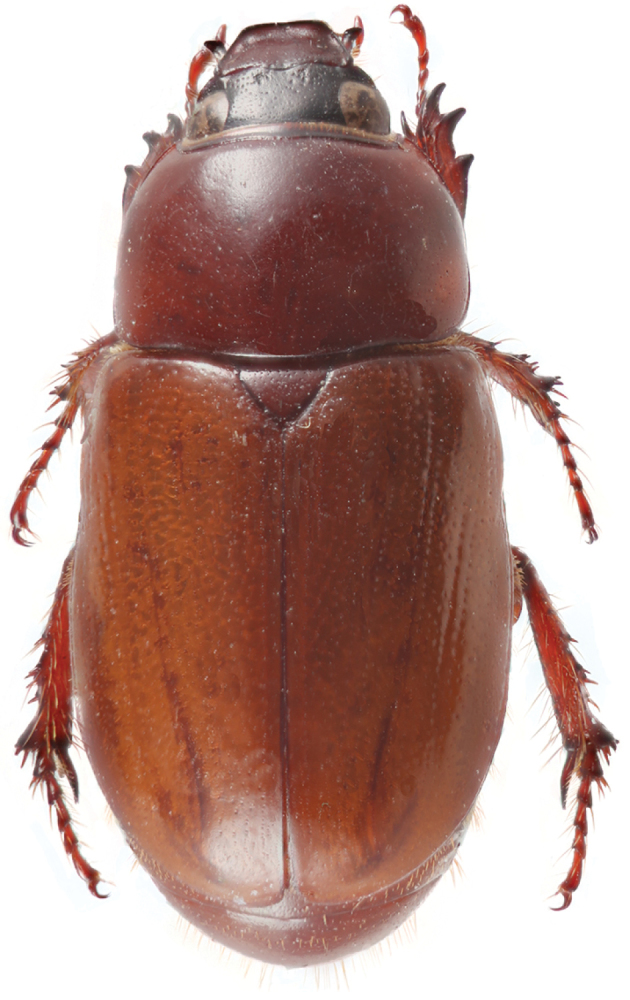
*Parapucayaamazonica* Prell.

**Figure 32.**
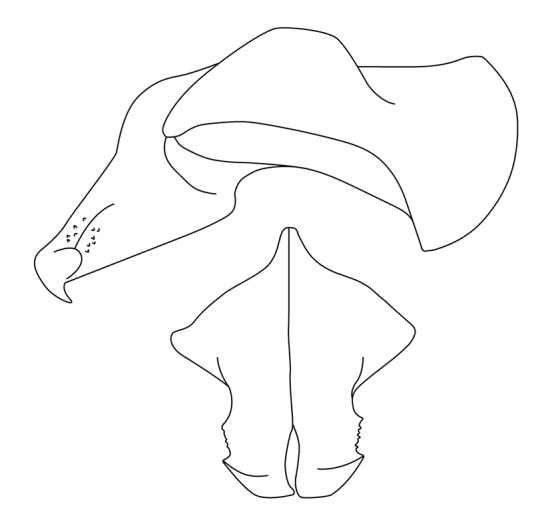
*Parapucayaamazonica* Prell parameres (from Ratcliffe 2003, by permission).

**Figure 33.**
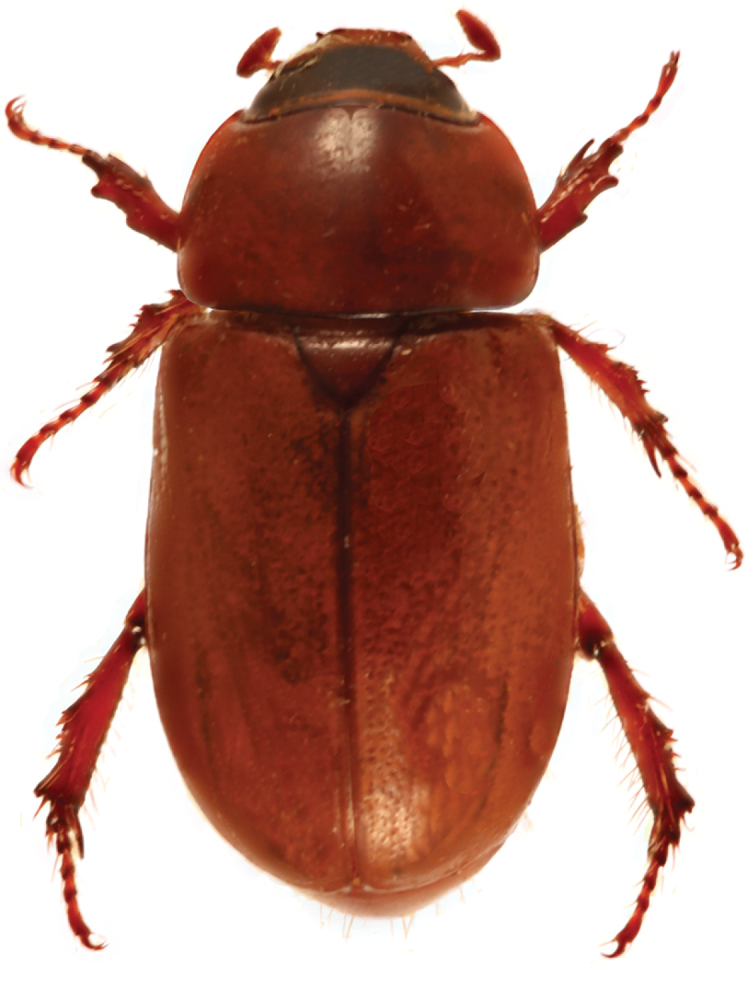
*Parapucayanodicollis* (Kirsch).

**Figure 34.**
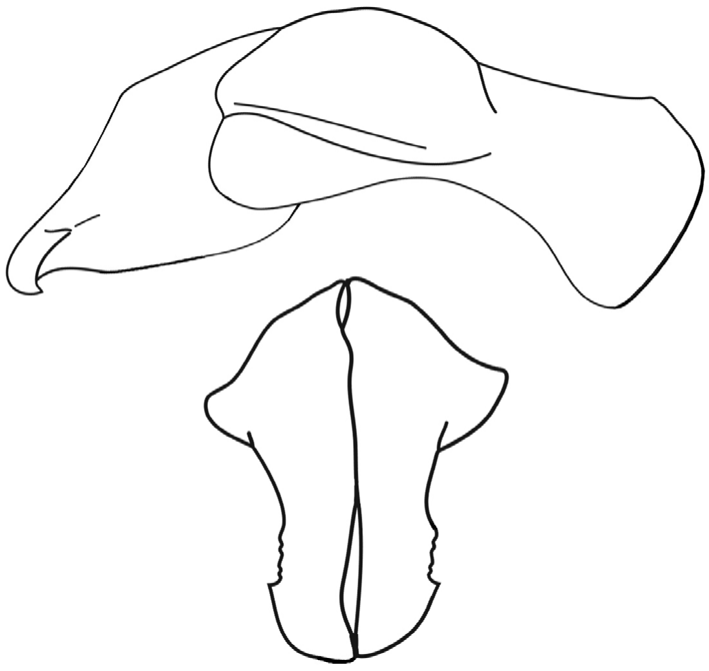
*Parapucayanodicollis* (Kirsch) parameres.

**Figures 35–36.**
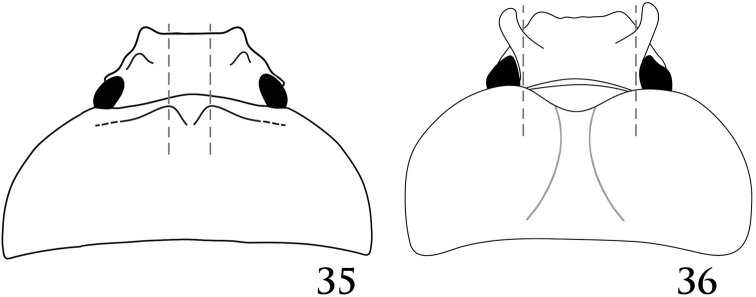
Pronota. **35***Pucayapulchra* Arrow **36***Pucayacastanea* Ohaus.

**Figure 37.**
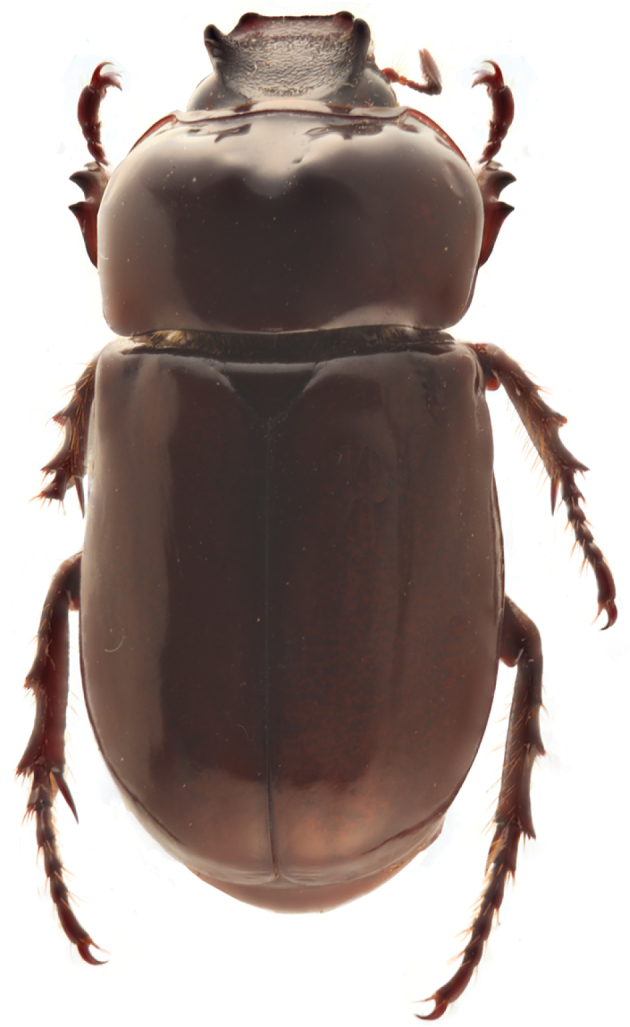
*Pucayacastanea* Ohaus.

**Figure 38.**
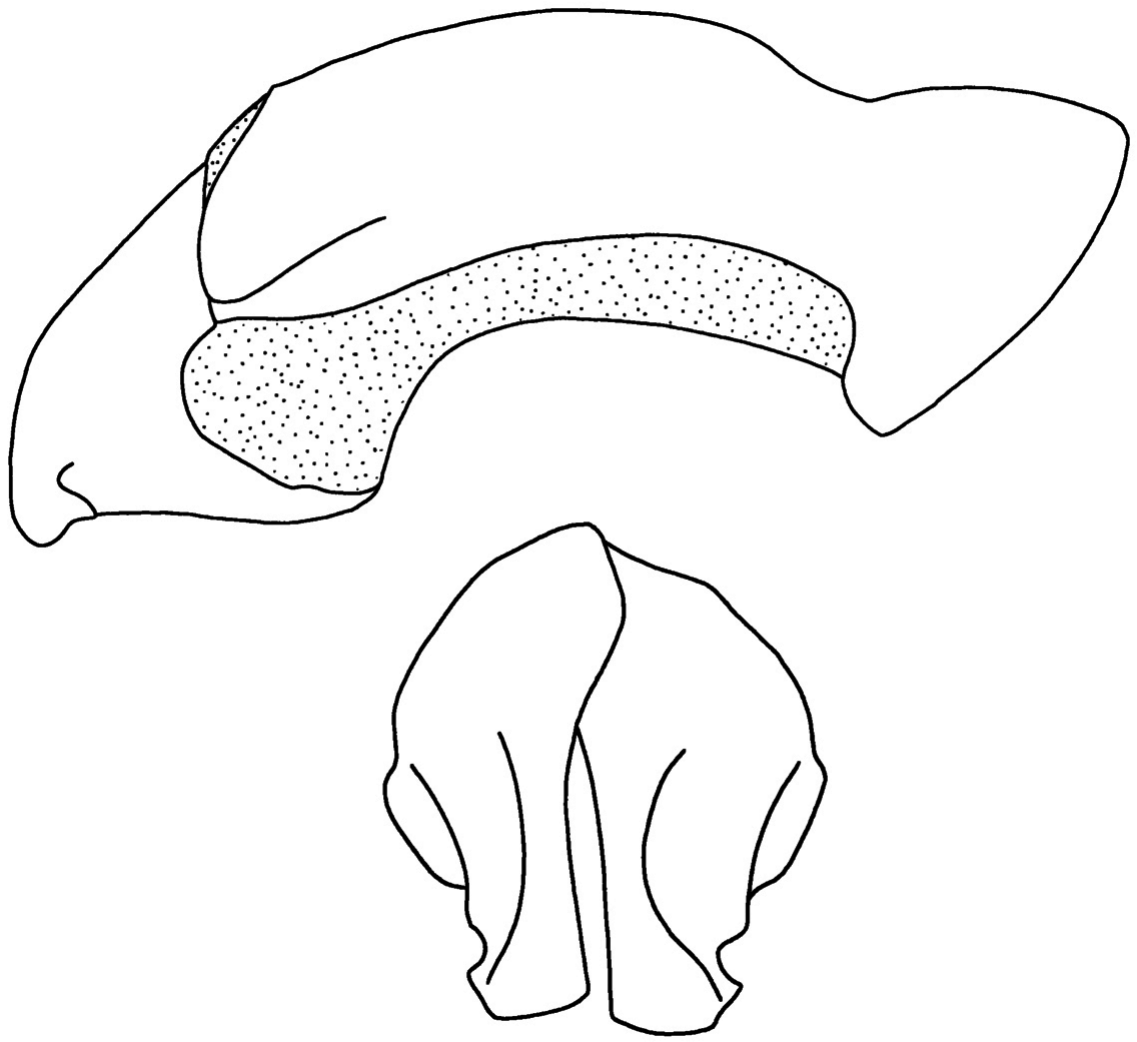
*Pucayacastanea* Ohaus parameres (from Ratcliffe 2003, by permission).

**Figure 39.**
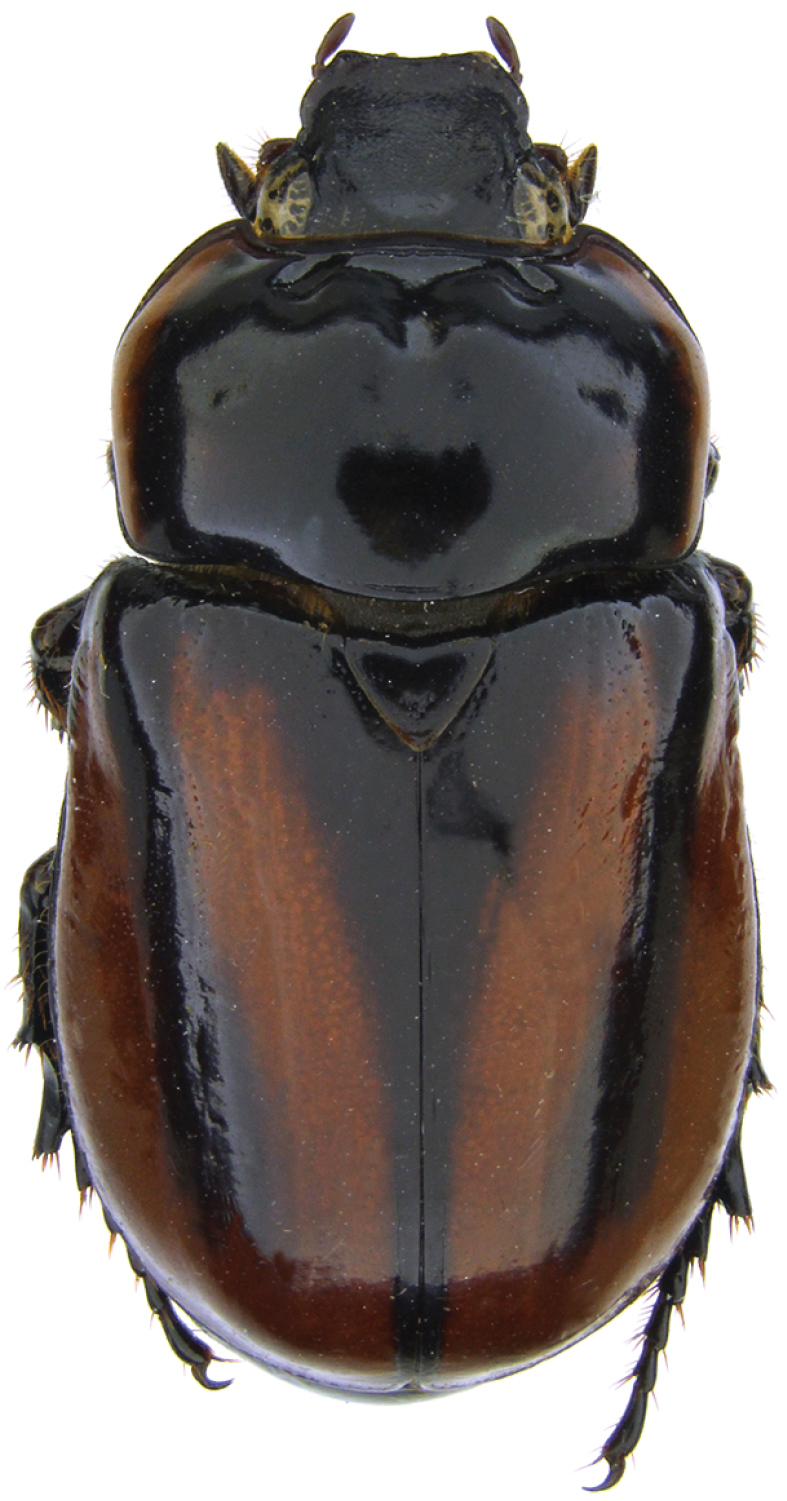
*Pucayapulchra* Arrow. Image by B.C. Ratcliffe.

**Figure 40.**
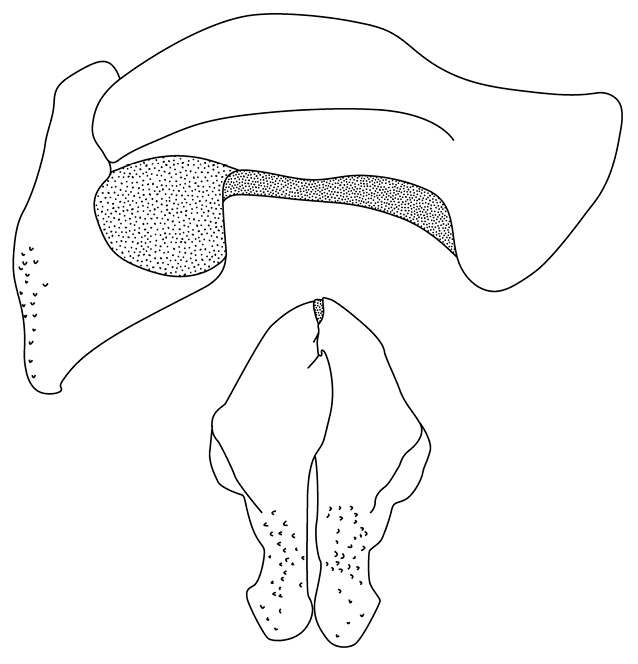
*Pucayapulchra* Arrow parameres.

#### Parapucaya

Taxon classificationAnimaliaColeopteraScarabaeidae

Prell, 1934

Figs 3 , 25 , 31 , 32 , 33 , 34

Parapucaya Prell, 1934: 162.

##### Notes.

*Parapucaya* contains two Neotropical species. The genus is distinct from other Cyclocephalini because of the presence of a strongly impressed frontoclypeal suture with the clypeus raised along the suture; lateral margin of clypeus near base raised into a subacute crest, evident in posterodorsal view (Fig. 25); and the presence of double tubercles or declivity near the anterior margin of the pronotum. It is necessary to look closely at the anterior margin of the pronotum to see the two small tubercles that help to characterize this genus, which can occasionally be difficult in some specimens where these tubercles are nearly absent, especially in *P.amazonica*. The color and general appearance of specimens of *Parapucaya* make them appear similar to *C.melanocephala* and other related species.

Adults of *Parapucaya* have been collected at lights at night. Species of this genus are found distributed in tropical lowlands, such as coastal and Amazonian rainforests, but also in areas with temperate climate, such as cloud forests. Based on label data of Ecuadorian individuals, specimens have been found in pastures. Nothing is known about the immature stages of *Parapucaya* species.

### Key to the species of *Parapucaya*

Males with protarsomeres enlarged, protarsus with one claw simple and one enlarged. Females with protarsomeres slender, protarsus with both claws simple.

**Table:** 

1	Pygidium glabrous. Male with round, minute, pronotal tubercle near mid-apex either side of midline; female with minute, transverse, pronotal tubercle near mid-apex either side of midline. Male parameres elongated (Fig. 34)	***P.nodicollis* (Kirsch)**
–	Pygidium setose around disc towards base. Male and female with barely perceptible declivity near mid-line of pronotal apex. Male parameres short (Fig. 32)	***P.amazonica* Prell**

#### Parapucaya amazonica

Taxon classificationAnimaliaColeopteraScarabaeidae

Prell, 1934

Figs 31 , 32

Parapucaya amazonica Prell, 1934: 162 (original combination).

##### Redescription.

Length 13.0–16.3 mm; width 6.3–8.0 mm. *Head*: Frons with sparse, small punctures, mostly on sides. Frontoclypeal suture complete, sinuate, deeply impressed. Clypeus sparsely, minutely punctate. Interocular width equals 2.7–3.0 transverse eye diameters. Antennal club subequal in length to antennomeres 2–7. *Pronotum*: Surface sparsely punctate; punctures minute on disc, small on sides. Two minute tubercles present just behind apex either side of midline, tubercles often reduced to a subapical declivity. *Elytra*: Surface with rows of small to moderate, ocellate punctures. *Pygidium*: Surface with disc sparsely punctate, punctures small. Base and lateral angles with moderately dense punctures; punctures small to moderate in size, base with transverse row of small setae appressed to surface (hence, difficult to see). In lateral view, surface strongly convex in males, weakly convex in females. *Legs*: Protibia strongly tridentate, basal tooth removed from other two teeth. Protarsus in males enlarged, larger claw strongly curved and incised at apex; females with protarsus simple. Metatibia with 7–8 short, thick spinules. *Venter*: Prosternal process moderate in length; apex transversely oval, with anterior 1/3–1/2 convex, posterior 2/3–1/2 flat. *Parameres*: Fig. 32.

##### Distribution.

*Parapucayaamazonica* is found from Costa Rica to Peru and Brazil (Endrődi 1969, 1985; Ratcliffe 2003). In Ecuador, it is widely distributed and has been recorded in thirteen provinces: Bolívar, Carchi, Cotopaxi, Esmeraldas, Loja, Los Ríos, Manabí, Napo, Orellana, Pastaza, Pichincha, Santo Domingo de los Tsáchilas, and Sucumbíos.

##### Diagnosis.

*Parapucayaamazonica* is invariably mistaken for species of *Cyclocephala* because of its similar appearance. The subapical declivity of the pronotum (or two tubercles in well-developed specimens), in combination with the raised basal margins of the clypeus and the raised clypeal surface along the frontoclypeal suture, will distinguish this genus from *Cyclocephala* species.

*Parapucayaamazonica* and *P.nodicollis* can be separated from each other by the shape of the mentum (concave from disc to apex in *P.amazonica*, evenly convex in *P.nodicollis*), the pronotal tubercles (subtle in *P.amazonica*, conspicuous in *P.nodicollis*), the presence or absence of pygidial setae (base and lateral angles of pygidium setose in *P.amazonica*, glabrous in *P.nodicollis*); size (in general, *P.amazonica* is larger and stouter than *P.nodicollis*, although some individuals overlap); and their parameres (Fig. 32).

##### Natural history.

In Ecuador, *P.amazonica* occurs at elevations ranging from sea level to 2,450 m in the coastal, Andean, and Amazon regions. Based on label data, adults can be collected throughout the year but in higher numbers in February and December. Nothing is known of the immature stages of this species.

#### Parapucaya nodicollis

Taxon classificationAnimaliaColeopteraScarabaeidae

(Kirsch, 1873)

Figs 33 , 34

Cyclocephala nodicollis Kirsch, 1873: 344 (original combination).

##### Redescription.

Length 11.8–13.0 mm; width 5.4–5.8 mm. *Head*: Frons rugulopunctate, punctures dense, moderate in size. Frontoclypeal suture complete, biarcuate. Clypeus subquadrate, surface rugo-punctate at base, shagreened at margins and disc; apex broadly truncate, slightly reflexed. Interocular width equals 2.5–3.0 transverse eye diameters. Antennal club slightly shorter than antennomeres 2–7. *Pronotum*: Surface moderately to densely punctate, punctures moderate in size, ocellate. *Pygidium*: Surface moderately to densely punctate, punctures moderate in size; glabrous. In lateral view, males with surface evenly rounded, females with surface nearly flat. *Legs*: Protibia tridentate, teeth subequally spaced. Protarsus in male weakly enlarged, median claw large, cleft at apex; protarsus and claw simple in female. *Venter*: Prosternal process moderately long, columnar; apex densely setose, flattened, and with large, raised, round “button” covering most of apex; setae long, tawny. *Parameres*: Fig. 34.

##### Distribution.

*Parapucayanodicollis* is known from Colombia, Ecuador, and Peru (Endrődi 1985). In Ecuador, it is recorded in five provinces in the coastal, Andean, and Amazonian regions: Esmeraldas, Morona Santiago, Napo, Pastaza, Pichincha, and Sucumbíos.

##### Diagnosis.

*Parapucayanodicollis* is usually mistaken for species of *Cyclocephala* because of its similar appearance. The two small tubercles on the pronotum, in combination with the raised basal margins of the clypeus and the raised clypeal surface along the frontoclypeal suture, will distinguish members of this genus from *Cyclocephala* species.

*Parapucayanodicollis* and *P.amazonica* can be separated from each other by the shape of the mentum (evenly convex in *P.nodicollis*, concave from disc to apex in *P.amazonica*); the pronotal tubercles (conspicuous in *P.nodicollis*, subtle in *P.amazonica*); the presence or absence of pygidial setae (glabrous in *P.nodicollis*, present across the base of the pygidium in *P.amazonica*); size (in general, *P.nodicollis* is smaller and thinner than *P.amazonica*, although some individuals overlap); and their parameres (Fig. 34).

##### Natural history.

In Ecuador, it occurs at elevations from 300 to 1,800 m on both sides of the Andes. Based on label data, adults can be collected in Ecuador throughout the year and in higher numbers in February, June to July, and in November. Nothing is known of the immature stages of this species.

#### Pucaya

Taxon classificationAnimaliaColeopteraScarabaeidae

Ohaus, 1910

Figs 4 , 6 , 35–36 , 37 , 38 , 39 , 40

Pucaya Ohaus, 1910: 675.

##### Notes.

The genus *Pucaya* contains two species, *P.castanea* Ohaus and *P.pulchra* Arrow. López-García et al. (2015) compared type specimens and synonymized *P.punctata* Endrődi with *P.pulchra* based on similarities in body length, pronotal and elytral punctation, and the fact that the description of *P.punctata* was based on the color and punctation of a single female in a species where color pattern and punctation are variable.

*Pucaya* is distinguished from other cyclocephalines by its broadly truncate clypeus that conceals the mandibles; a small horn or tubercle near each eye (horns not as developed in Ecuadorian specimens as in Panamanian specimens); parameres with round, minute spinules (bumps) on the apical half; and a characteristic binodose pronotum.

Specimens can be taken at light traps, and some have been collected with pitfall traps. In Ecuador, species of this genus are widely distributed as follows: Chocó region in the coast; premontane, montane, and cloud forests in the Andean region; and rainforests in the Amazon basin. Life history information is lacking.

### Key to the species of *Pucaya*

**Table:** 

1	Elytra with impressed sutural stria at least on apical half. Protuberances on pronotum with their highest points close to midline, positioned between frontoclypeal tubercles in posterodorsal view (Fig. 35). Parameres as in Fig. 40	***P.pulchra* Arrow**
–	Elytra without impressed sutural stria. Nodes on pronotum evenly round, widely separated, with their highest points about “in-line” with frontoclypeal tubercles in posterodorsal view (Fig. 36). Parameres as in Fig. 38	***P.castanea* Ohaus**

#### Pucaya castanea

Taxon classificationAnimaliaColeopteraScarabaeidae

Ohaus, 1910

Figs 36 , 37 , 38

Pucaya castanea Ohaus, 1910: 676 (original combination).
Pucaya columbiana Beck, 1942: 47 (synonym).

##### Redescription.

Length 24.0–30.1 mm; width 11.0–14.2 mm. Color light to dark reddish brown; head, tibiae, and tarsi often black. *Head*: Frons and clypeus completely rugulose in males, partially rugulose to nearly smooth in females. Base of clypeus at sides (and just in front of eye) with short, vertically upright horn in males or a large tubercle in females. Clypeus with apex very broadly truncate, shallowly emarginate, broadly reflexed in males, narrowly reflexed in females. Interocular width equals 5.0 transverse eye diameters. Antenna with 10 antennomeres, club subequal to antennomeres 2–7. Mandibles small, narrow, not visible in dorsal view. *Pronotum*: Surface with sparse, minute punctures. A tumescent boss present either side of broadly depressed midline. Narrow marginal bead present on base. *Elytra*: Surface also with sparse, minute punctures; punctures becoming denser along lateral margins. Striae totally lacking. *Pygidium*: Surface with sparse, minute punctures. In lateral view, regularly convex in males, nearly flat in females. *Legs*: Protibia tridentate, basal tooth slightly removed from others. Males with claw of anterior tarsus enlarged, apex split. Apex of posterior tibia arcuate and with 9 short, stout spinules. Apex of first tarsomere of posterior tarsus triangularly elongated. *Venter*: Prosternal process short; apex transversely oval, anterior 1/2–2/3 convex, posterior 1/2–1/3 flat, a transverse sulcus often separating anterior and posterior parts. *Parameres*: Fig. 38.

##### Distribution.

*Pucayacastanea* occurs in Costa Rica, Panama, Colombia, and Ecuador (Beck 1942b; Endrődi 1969, 1985; Ratcliffe 2003; López-García et al. 2015). In Ecuador, it is widely distributed in thirteen provinces: Azuay, Cañar, Carchi, Cotopaxi, Esmeraldas, Imbabura, Loja, Morona Santiago, Napo, Orellana, Pichincha, Santo Domingo de los Tsáchilas, and Zamora Chinchipe.

##### Diagnosis.

*Pucayacastanea* can be distinguished from *P.pulchra* by its elytral punctation. In *P.castanea*, the entire elytral surface has sparse, minute punctures, while in *P.pulchra* the elytral surface is striate-punctate from the base to 2/3 the length of the elytra. The punctures are dense, moderate in size, and ocellate, but on the apical third of the elytra the punctures are sparse and minute. The form of the parameres (Fig. 38) also separates both species.

##### Natural history.

In Ecuador, *P.castanea* occurs at elevations ranging from sea level to 2,550 m in the coastal, Andean, and Amazon regions. Based on label data, adults can be collected throughout the year but in higher numbers from February to May and from November to December. Nothing is known of the immature stages of this species.

#### Pucaya pulchra

Taxon classificationAnimaliaColeopteraScarabaeidae

Arrow, 1911

Figs 6 , 35 , 39 , 40

Pucaya pulchra Arrow, 1911: 167 (original combination).

##### Redescription.

Length 20.4–23.7 mm; width 9.8–11.2 mm. Color of head black or piceous. Pronotum completely black or black with brown, elongate markings on margins, with or without brown spots on base of disc. Elytra entirely black or black with brown margins or brown with black markings on the suture, humerus and behind scutellum; markings can be short near elytral base or extend to umbone area. Scutellum, pygidium, venter, and legs black or brown. *Head*: Frons sparsely punctate at base, becoming progressively rugo-punctate anteriorly; punctures moderate in size. Frontoclypeal sutural area at sides with tubercle in both sexes; tubercle smaller in females, conical in males. Clypeus with apex very broadly truncate, reflexed, surface rugose at disc, smooth to shagreened at margins. Interocular width equals 4.1–4.3 transverse eye diameters. Antenna with 10 antennomeres, club slightly longer than antennomeres 2–7. *Pronotum*: Surface moderately to densely punctate at base, punctures moderate in size; sparsely punctate from disc to apex, punctures minute. Broadly depressed midline, with round depressions on each side of midline: 1 on apex, 2 between mid-disc and margins; depressions shallow in females. *Elytra*: Surface from base to 2/3 striate-punctate; punctures dense, moderate in size, ocellate; from 2/3 to apex with sparse, minute punctures. *Pygidium*: Surface densely punctate, punctures moderate in size. In lateral view, males with surface evenly rounded, females with surface nearly flat. *Legs*: Protibia tridentate. Protarsus in male weakly enlarged, median claw large, strongly curved, cleft at apex; protarsus and claw simple in female. *Venter*: Prosternal process moderately long, columnar; apex densely setose, flattened, and with large, raised, round “button” covering most of apex; setae long, tawny. *Parameres*: Fig. 40.

##### Distribution.

*Pucayapulchra* occurs in Colombia and Ecuador (Endrődi 1985; López-García et al. 2015). In Ecuador, adults were collected in five provinces: Esmeraldas, Loja, Napo, Pastaza, Pichincha, Santo Domingo de los Tsáchilas, Tungurahua, and Zamora Chinchipe.

##### Diagnosis.

*Pucayapulchra* can be distinguished from *P.castanea* by the elytral punctation . In *P.pulchra*, the elytral surface is striate-punctate from the base to 2/3 of the elytra; the punctures are dense, moderate in size, and ocellate, but the apical third has sparse, minute punctures. In *P.castanea*, the entire elytral surface has sparse, minute punctures. The form of the parameres (Fig. 40.) also separates both species.

##### Natural history.

In Ecuador, *P.pulchra* occurs at elevations ranging from 20 to 1,900 m in the coastal, Andean, and Amazon regions. Some specimens have been collected in pitfall traps.

## Supplementary Material

XML Treatment for Parapucaya

XML Treatment for Parapucaya amazonica

XML Treatment for Parapucaya nodicollis

XML Treatment for Pucaya

XML Treatment for Pucaya castanea

XML Treatment for Pucaya pulchra

## References

[B1] Ahrens D Schwarzer J Vogler AP (2014) The evolution of scarab beetles tracks the sequential rise of angiosperms and mammals. Proceedings of the Royal Society B 281(1791): e20141470. 10.1098/rspb.2014.1470 PMC4132691 25100705

[B2] Ahrens D Scott M Vogler AP (2011) The phylogeny of monkey beetles based on mitochondrial and ribosomal RNA genes (Coleoptera: Scarabaeidae: Hopliini). Molecular Phylogenetics and Evolution 60: 408–415. 10.1016/j.ympev.2011.04.011 21571081

[B3] Arnett RH (1973) The beetles of the United States. A manual for identification. American Entomological Institute, Ann Arbor, 387–438.

[B4] Arrow GJ (1911) Notes on the coleopterous subfamily Dynastinae, with descriptions of new genera and species. Annals and Magazine of Natural History (series 8) 8: 151–176. 10.1080/00222931108693008

[B5] Arrow GJ (1937) Coleopterorum Catalogus, pars 156. Scarabaeidae: Dynastinae. W Junk, Berlin, 124 pp.

[B6] Bates HW (1888) Pectinicornia and Lamellicornia, Family Dynastidae In: Godman FD, Salvin O (Eds) Biologia Centrali-Americana – Insecta, Coleoptera, Volume 2, Part 2. 296–342.

[B7] Beach JH (1982) Beetle pollination of *Cyclanthusbipartitus* (Cyclanthaceae). American Journal of Botany 69: 1074–1081. 10.1002/j.1537-2197.1982.tb13352.x

[B8] Beach JH (1984) The reproductive biology of the peach or “pejibaye” palm (*Bactrisgasipaes*) and a wild congener (*B.porschiana*) in the Atlantic lowlands of Costa Rica. Principes 28: 107–119.

[B9] Beck P (1942) Description d’une variété nouvelle de *Pucayacastanea*. Bulletin de la Societe entomologique de France 47(3–4): 47–48.

[B10] Blackwelder RE (1944) Checklist of the coleopterous insects of Mexico, Central America, the West Indies, and South America, part 2. Bulletin of the U. S. National Museum 185: 189–341.

[B11] Casey TL (1915) A review of the American species of Rutelinae, Dynastinae, and Cetoniinae. Memoirs of the Coleoptera 6: 1–394.

[B12] Clark DR (2011) Phylogenetic analysis of the scarab beetle tribe Cyclocephalini (Coleoptera: Scarabaeidae: Dynastinae) based on adult morphological characters. MSc Thesis, Department of Biological Sciences, Wichita State University, Wichita, 68 pp.

[B13] Cramer JM Meese ADJ Tuenissen PA (1975) A note on the pollination of nocturnally flowering species of *Nymphaea*. Acta Botanica Neerlandica 24: 489–490. 10.1111/j.1438-8677.1975.tb01039.x

[B14] Dechambre R-P (2006) Nouveaux dynastides néotropicaux (Coleoptera, Dynastidae). Coléopteres 12(17): 227–233.

[B15] Dieringer G Cabrera RL Lara M Loya L Reyes-Castillo P (1999) Beetles pollination and floral thermogenicity in *Magnoliatamaulipana* (Magnoliaceae). International Journal of Plant Sciences 160: 64–71. 10.1086/314099

[B16] Endrődi S (1969) Monographie der Dynastinae 4. Tribus: Pentodontini (Coleoptera, Lamellicornia). Entomologische Abhandlungen 87: 1–145.

[B17] Endrődi S (1985) The Dynastinae of the World. Dr. W. Junk Publ., Dordrecht, 800 pp.

[B18] Gasca-Álvarez HJ Vasconcelos da Fonseca CR Ratcliffe BC (2008) Synopsis of the Oryctini (Coleoptera: Scarabaeidae: Dynastinae) from the Brazilian Amazon. Insecta Mundi 61: 1–62.

[B19] Gottsberger G (1989) Beetle pollination and flowering rhythm of *Annona* spp. (Annonaceae) in Brazil. Plant Systematics and Evolution 167: 165–187. 10.1007/BF00936404

[B20] Gottsberger G Silberbauer‐Gottsberger I Seymour RS Dötterl S (2012) Pollination ecology of *Magnoliaovata* may explain the overall large flower size of the genus. Flora – Morphology, Distribution, Functional Ecology of Plants 207: 107–118. 10.1016/j.flora.2011.11.003

[B21] Gunter NL Weir TA Slipinski A Bocak L Cameron SL (2016) If dung beetles (Scarabaeidae: Scarabaeinae) arose in association with dinosaurs, did they also suffer a mass extinction at the K-Pg boundary? PLOS ONE 11(5): e0153570. 10.1371/journal.pone.0153570 PMC4856399 27145126

[B22] Hirthe G Porembski S (2003) Pollination of *Nymphaealotus* (Nymphaeaceae) by rhinoceros beetles and bees in the northeastern Ivory Coast. Plant Biology 5: 670–676. 10.1055/s-2003-44717

[B23] Hoang DT Chernomor O von Haeseler A Minh BQ Vinh LS (2017) UFBoot2: improving the ultrafast bootstrap approximation. Molecular Biology and Evolution 35: 518–522. 10.1093/molbev/msx281 PMC5850222 29077904

[B24] Hoang DT Vinh LS Flouri T Stamatakis A von Haeseler A Minh BQ (2018) MPBoot: fast phylogenetic maximum parsimony tree inference and bootstrap approximation. BMC Evolutionary Biology 18: 1–11. 10.1186/s12862-018-1131-3 29390973 PMC5796505

[B25] Jameson ML (1998) Generic Guide to the New World Scarab Beetles. Scarabaeidae (Latreille 1802). University of Nebraska State Museum, Lincoln, Nebraska. http://museum.unl.edu/research/entomology/Guide/Scarabaeoidea/Scarabaeidae/Scarabaeidae-pages/Scarabaeidae-Overview/ScarabaeidaeO.html

[B26] Jameson ML Jakl S (2010) Synopsis of the aroid scarabs in the genus *Peltonotus* Burmeister (Scarabaeidae: Dynastinae: Cyclocephalini) from Sumatra and description of a new species. ZooKeys 34: 141–152. 10.3897/zookeys.34.302

[B27] Jameson ML Ratcliffe BC Maly V (2002) Review of the genus *Acrobolbia* Ohaus with remarks on its classification and key to the world genera of Cyclocephalini (Coleoptera: Scarabaeidae: Dynastinae). Folia Heyrovskyana 10: 1–15.

[B28] Kalyaanamoorthy S Minh BQ Wong TKF von Haeseler A Jermiin LS (2017) ModelFinder: fast model selection for accurate phylogenetic estimates. Nature Methods 14: 587–589. 10.1038/nmeth.4285 28481363 PMC5453245

[B29] Kearse M Moir R Wilson A Stones-Havas S Cheung M Sturrock S Buxton S Cooper A Markowitz S Duran C Thierer T Ashton B Mentjies P Drummond A (2012) Geneious Basic: an integrated and extendable desktop software platform for the organization and analysis of sequence data. Bioinformatics 28: 1647–1649. 10.1093/bioinformatics/bts199 22543367 PMC3371832

[B30] Kirsch T (1873) Beiträge zur Kenntniss der peruanische Käferfauna I–II. Berliner Entomologische Zeitschrift 17: 121–152.

[B31] Kumar S Stecher G Tamura K (2016) MEGA7: Molecular evolutionary genetics analysis version 7.0 for bigger datasets. Molecular Biology and Evolution 33: 1870–1874. 10.1093/molbev/msw054 27004904 PMC8210823

[B32] Lanfear R Frandsen PB Wright AM Senfeld T Calcott B (2016) PartitionFinder 2: New methods for selecting partitioned models of evolution for molecular and morphological phylogenetic analyses. Molecular Biology and Evolution 34: 772–773. 10.1093/molbev/msw260 28013191

[B33] Larkin MA Blackshields G Brown NP Chenna R McGettigan PA McWilliam H Valentin F Wallace IM Wilm A Lope R Thompson JD Gibson TJ Higgins DG (2007) ClustalW and ClustalX version 2.0. Bioinformatics 23: 2947–2948. 10.1093/bioinformatics/btm404 17846036

[B34] Leng CW (1920) Catalogue of the Coleoptera of America, North of Mexico. John D. Sherman, Mt. Vernon, NY, 470 pp.

[B35] López-García MM Gasca-Álvarez HJ Amat-García G (2015) The scarab beetle tribe Pentodontini (Coleoptera: Scarabaeidae: Dynastinae) of Colombia: taxonomy, biology and distribution. Zootaxa 4048(4): 451–492. 10.11646/zootaxa.4048.4.1 26624762

[B36] Moore MR (2012) A new female elytral character for the tribe Cyclocephalini (Coleoptera: Scarabaeidae: Dynastinae) and an observation of its possible function. The Coleopterists Bulletin 66: 200–202. 10.11646/zootaxa.4048.4.1

[B37] Moore MR Beza-Beza CF Wickell DA Beck JB Jameson ML (2015) Molecules, Morphology and *Mimeoma* Scarabs: Evolutionary and Taxonomic Implications for A Palm-Associated Scarab Group. Systematic Entomology 40(4): 891–900 10.1111/Syen.12139

[B38] Moore MR Cave RD Branham MA (2018a) Synopsis of the cyclocephaline scarab beetles (Coleoptera, Scarabaeidae, Dynastinae). ZooKeys 745: 1–99. 10.3897/zookeys.745.23683 PMC5904508 29670448

[B39] Moore MR Cave RD Branham MA (2018b) Annotated catalog and bibliography of the cyclocephaline scarab beetles (Coleoptera, Scarabaeidae, Dynastinae, Cyclocephalini). ZooKeys 745: 101–378. 10.3897/zookeys.745.23685 PMC5904534 29670449

[B40] Moore MR Jameson ML (2013) Floral associations of cyclocephaline scarab beetles. Journal of Insect Science 13(100): 1–43. 10.1673/031.013.10001 24738782 PMC4062068

[B41] Morón MA Ratcliffe BC (1996) New tribal placement of the genus *Coscinocephalus* Prell, 1936, with description of the larva, pupa and adult of a new species from Mexico (Coleoptera: Scarabaeoidea; Dynastinae). Journal of the New York Entomological Society 104: 48–61.

[B42] Mulsant E (1842) Histoire Naturelle des Coléoptères de France, pt. 2. Lamellicornes. Maison, Paris, 623 pp.

[B43] Ohaus F (1910) Neue südamerikanische Dynastiden (Col.). Deutsche Entomologische Zeitschrift 1910: 671–690.

[B44] Palumbi S Martin A Romano S McMillan WO Stice L Grabowski G (1991) The simple fool’s guide to PCR. Version 2.0. Department of Zoology and Kewalo Marine Laboratory, University of Hawaii, Honolulu, 45 pp.

[B45] Prell H (1934) Beiträge zur Kenntnis der Dynastinen (XII). Beschreibungen und Bemerkungen. Entomologische Zeitschrift 47: 162–164.

[B46] Rambaut A Drummond AJ (2013) Tracer v.1.6. http://tree.bio.ed.ac.uk/software/tracer/

[B47] Ratcliffe BC (2003) The dynastine scarab beetles of Costa Rica and Panama (Coleoptera: Scarabaeidae: Dynastinae). Bulletin of the University of Nebraska State Museum 16: 1–506.

[B48] Ratcliffe BC Cave RD (2015) The dynastine scarab beetles of the West Indies (Coleoptera: Scarabaeidae). Bulletin of the University of Nebraska State Museum 28: 1–346.

[B49] Ratcliffe BC Cave RD (2017) The dynastine scarab beetles of the United States and Canada (Coleoptera: Scarabaeidae). Bulletin of the University of Nebraska State Museum 30: 1–298.

[B50] Ratcliffe BC Morón MA (1997) Dynastinae. In: Morón MA Ratcliffe BC Deloya C (Eds) Atlas de los Escarabajos de México.Coleoptera: Lamellicornia. Vol. 1. Familia *Melolonthidae*. Comisión Nacional para el Conocimiento y Uso de la Biodiversidad (CONABIO) and Sociedad Mexicana de Entomologia, Mexico, D. F., 53–98.

[B51] Ronquist F Huelsenbeck JP (2003) MRBAYES 3: Bayesian phylogenetic inference under mixed models. Bioinformatics 19: 1572–1574. 10.1093/bioinformatics/btg180 12912839

[B52] Sanabria-García R Gasca-Álvarez HJ Amat-García G (2012) Sinopsis de la Tribu Oryctini (Coleoptera: Scarabaeidae: Dynastinae) de Colombia. Insecta Mundi 276: 1–64.

[B53] Smith ABT (2006) A review of the family‐group names for the superfamily Scarabaeoidea (Coleoptera) with corrections to nomenclature and a current classification. The Coleopterists Bulletin 60: 144–204. 10.1649/0010-065X(2006)60[144:AROTFN]2.0.CO;2

[B54] Trifinopoulos J Nguyen L-T von Haeseler A Minh BQ (2016) W-IQ-TREE: a fast online phylogenetic tool for maximum likelihood analysis. Nucleic Acids Research 44: W232–W235. 10.1093/nar/gkw256 PMC4987875 27084950

[B55] Whiting MF (2001) Mecoptera is paraphyletic: multiple genes andphylogeny of Mecoptera and Siphonaptera. Zoologica Scripta 31: 93–104. 10.1046/j.0300-3256.2001.00095.x

[B56] Whiting MF Carpenter JC Wheeler QD Wheeler WC (1997) The Strepsiptera problem: phylogeny of the holometabolous insect orders inferred from 18S and 28S ribosomal DNA sequences and morphology. Systematic Biology 46: 1–68. 10.1093/sysbio/46.1.1 11975347

[B57] Young HJ (1986) Beetle pollination of *Dieffenbachialongispatha* (Araceae). American Journal of Botany, 73: 931–944. 10.1002/j.1537-2197.1986.tb12133.x

[B58] Young HJ (1988) Neighborhood size in a beetle pollinated tropical aroid: effects of low density and asynchronous flowering. Oecologia 76: 461–466. 10.1007/BF00377043 28312028

